# Reaction of a Laser-Ablated Al Plume with a Fluorinated Ionic-Liquid Surface: Characterizing the AlF-Producing Species

**DOI:** 10.1021/acs.jpcc.6c03846

**Published:** 2026-08-07

**Authors:** Philip A. J. Pearcy, Paul. D. Lane, Peter T. Rubli, Christian T. Haakansson, Peter D. Watson, Stuart R. Mackenzie, Naomi S. Elstone, Duncan. W. Bruce, John M. Slattery, Matthew L. Costen, Kenneth G. McKendrick

**Affiliations:** † Institute of Chemical Sciences, School of Engineering and Physical Sciences, 3120 Heriot-Watt University, Edinburgh EH14 4AS, U.K.; ‡ Department of Chemistry, Chemistry Research Laboratory, 6396 University of Oxford, Mansfield Rd, Oxford OX1 3TA, U.K.; § Department of Chemistry, University of York, Heslington, York YO10 5DD, U.K.

## Abstract

The species present in a laser-ablated Al plume have been characterized and their reactivity with the surface of the ionic liquid, 1-ethyl-3-methylimidazolium bis­(trifluoromethylsulfonyl)­imide ([C_2_mim]­[Tf_2_N]), has been investigated. Al atoms were confirmed to be present and their kinetic-energy distributions determined by laser-induced fluorescence time-of-flight measurements, over a wider range of ablation fluences than reported previously. Cations were detected directly using an in-line microchannel-plate-detector assembly. Corroboratory measurements of the ions entrained in a He buffer gas confirmed Al^+^ to be the only cation present in measurable concentrations. The Al^+^ kinetic energies were determined by time-of-flight and found to be much hotter than anticipated based on previous independent reports; under the highest-fluence conditions examined (30 mJ pulse^–1^, nominal fluence of 42 J cm^–2^), the mean kinetic energies of Al and Al^+^ were 17 and 450 eV, respectively. Using an electrostatic deflector, it was shown that both Al and Al^+^ projectiles produce AlF through reaction at the surface of the ionic liquid [C_2_mim]­[Tf_2_N] containing a fluorinated anion. The AlF yield from Al^+^ is mildly dominant under our conditions. AlF leaves the surface with near-thermal kinetic energy and rotational distributions. There is a minor component of vibrationally excited AlF which is confined to the fastest products. The observation of near-complete thermalization of the AlF products is consistent with significant penetration of the projectiles into the liquid, as would be anticipated from the high incident kinetic energies, particularly for Al^+^. The results provide new insights into reactive-atom scattering (RAS) from fluorine-containing liquid surfaces.

## Graphical abstract

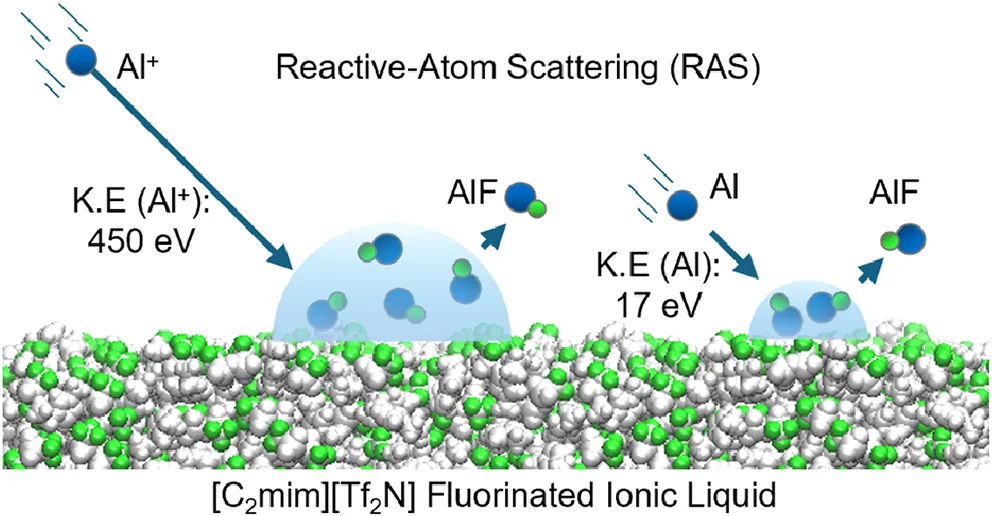

## Introduction

Laser ablation is a very well-established method for the volatilization of metallic elements.^1,2^ It has been used to produce gas-phase atoms, clusters and complexes in a range of charge states, enabling a wide range of studies in areas including spectroscopy and structure determination.^3–9^ These applications include the production of atomic or ion beams with defined kinetic-energy (KE) distributions for fundamental investigations of chemical reactivity.^10–13^ A novel extension of this approach, which we have introduced recently and pursue further here,^14,15^ is to generate probe projectiles for a new variant of a surface-analytical technique known as reactive-atom scattering (RAS).^16–28^

In essence, RAS is based on selecting a suitable probe projectile that generates only a specific product in reaction with particular functional groups at the surface of the material of interest. The specific product is detected in the gas phase above the material, in practice so far either by mass spectrometry (RAS-MS) as developed by Minton and co-workers,^18,20–23,26,27,29^ or by laser-induced fluorescence (RAS-LIF) as introduced in parallel by our own group.^16,17,19–21,23–26,28^

The original RAS probe projectiles were either primarily O or, in a few cases, F atoms. They proved successful in measuring the surface exposure of alkyl groups in a wide range of ionic liquids (ILs) and their mixtures, through selective H-abstraction reactions.^16–28^ The IL targets of these studies are themselves of widespread interest because they offer a unique combination of properties, such as low vapor pressure, thermal stability, solvation versatility, and broad electrochemical windows that have been exploited in a broad range of applications.^30–36^ Their surface structures are particularly relevant in processes such as gas separation and multiphase catalysis where gas-phase molecules are accommodated at, and transported through, the gas–liquid interface.^31,34^

The most-recent version of RAS-LIF exploits laser ablation of solid Al to produce a reactive plume that generates gas-phase AlF from fluorinated groups in the target material.^14,15^ We have demonstrated that AlF is produced from a range of fluorine-containing materials, including solid polytetrafluoroethylene (PTFE) and liquid perfluoropolyether (PFPE), but our primary focus has remained on ILs that incorporate fluorine-containing functional groups.^14,15^ This is motivated partly by the general fact that many of the anions in widespread use in ILs contain fluorine. More specifically, based on the well-known differences in molecular-level properties (volume, stiffness, polarity, and polarizability) between alkyl and fluoroalkyl chains, it has been postulated that IL mixtures composed of cations with these two chain types may provide a route to efficient tailoring of properties for particular applications, such as multiphase catalysis.^28,37–48^

In our previous exploratory work, we have shown that AlF is produced when an Al-ablation plume interacts with ILs containing three different fluorinated anions: bis­(trifluoromethylsulfonyl)­imide, [Tf_2_N]^−^; trifluoromethanesulfonate, [OTf]^−^; and tetrafluoroborate, [BF_4_]^−^.^15^ These anions were chosen because they are representative of those in common use and span a range of F atom chemical environments. They were combined with cations from the well-known 1-alkyl-3-methylimidazolium family, with two different alkyl chain lengths: 1-ethyl-3-methylimidazolium ([C_2_mim]^+^) and 1-octyl-3-methylimidazolium ([C_8_mim]^+^).

Although some of the expected qualitative correlations were observed, such as the general drop in AlF yield for longer alkyl chains that is consistent with a reduction in F atom surface exposure due to greater overshadowing, there were a number of surprises.^15^ In general, the AlF yields did not quantitatively reproduce the predicted “outer-surface-exposures” of F atoms for different ILs; these are rigorously defined quantities obtained by applying a combination of ball-drop and solvent-accessible-surface-area methods to the results of molecular dynamics (MD) simulations.^15^ More specifically, the yields of AlF from [OTf]^−^-containing ILs were anomalously low relative to the other two anions, which were roughly equal. This is unexpected because of the apparently greater chemical similarity of the F atom environments in [Tf_2_N]^−^ and [OTf]^−^, which both differ significantly from [BF_4_]^−^.

This has led us to the current work, in which we seek to better understand some of the underlying reasons for these, and other related, observations. In particular, we aim to further determine the speciation of the projectiles in the Al-ablation plume and to characterize their KE distributions. We had previously demonstrated directly by LIF detection that ground-state Al atoms were generated.^14^ Time-of-flight (TOF) measurements showed that under mild, but typical, ablation conditions, the KE distribution was approximately Maxwellian, with a characteristic temperature of ∼45,000 K, a most-probable KE of 187 kJ mol^–1^ (1.9 eV), and a mean of 560 kJ mol^–1^ (5.8 eV). This is considerably colder than previous independent measurements of Al ablation using mass-spectrometric methods with secondary ionization of neutral species.^49^ Moreover, Torrisi et al. had also reported significant production of cations, dominated by Al^+^ at lower ablation fluences, with a minor contribution from higher charge states that increased at higher fluences. The KE distributions of the ions were progressively hotter with increasing charge state.^49,50^

The KE distribution is clearly an important factor affecting the penetration depth and hence surface-sensitivity of RAS as an analytical method. However, it is not the only one: as we have emphasized, RAS belongs to a general family of surface-sensitive methods in which a projectile (generally defined) interacts with the surface and a reporter species is detected.^15^ Other examples in this category include angle-resolved photoelectron spectroscopy (ARXPS),^51–54^ Rutherford backscattering (RBS);^55–57^ low-energy ion scattering (LEIS);^58–60^ neutral-impact-collision ion-scattering spectroscopy (NICISS);^61–64^ secondary-ion mass spectrometry (SIMS);^57,65^ metastable-atom electron spectroscopy (MAES)/metastable-induced electron spectroscopy (MIES);^64,66,67^ and reactive-ion scattering (RIS).^68,69^ Many of these methods are based on higher-KE projectiles than the Al atoms in our own previous experiments,^14^ or the Al^+^ ions reported by Torrisi et al.,^49^ but that does not in itself prevent them from being surface-sensitive. This further depends on whether the incident projectile only survives to a shallow depth, as in MAES/MIES, or whether the reporter species is only capable of escaping from a shallow depth (either by avoiding being lost completely, or with its own KE distribution unmodified and hence capable of being distinguished), as in many of the others. Nevertheless, knowing the KE distributions of the projectile(s) represents a very important step toward understanding the interaction of the Al plume with the IL surfaces in this version of the RAS-LIF method.

We report here modifications to our RAS-LIF apparatus that allow direct measurements of ion production in the ablation process and of their KE distributions as a function of ablation fluence. The dominant ion present is demonstrated to be Al^+^ in corroboratory experiments, in which the initial KE is quenched in a He carrier gas, under otherwise similar ablation conditions. An electrostatic deflector is introduced to determine the separate contributions of Al and Al^+^ to AlF production from a prototypical IL containing a fluorinated anion, [C_2_mim] [Tf_2_N]. The KE distributions of the AlF products recoiling from the surface produced by each of Al and Al^+^ are characterized separately. We reflect on the implications of the results for this version of RAS-LIF as a surface-analytical method.

## Experimental Methods

The majority of the experiments were conducted in a modified version of the RAS-LIF apparatus described in previous papers.^14,15^ In summary, the established elements of the apparatus, included in the schematic diagram in Figure 1a, were a moveable Al metal rod in vacuum, which was irradiated by a focused pulse of laser light. The ablated plume passed through a series of apertures, forming a beam of projectiles that propagated along the main axis of the chamber. This beam impinged on the liquid surface, created by rotating a stainless-steel wheel in a bath of the liquid of interest. The incident beam, and the products of any reaction, were intercepted a short distance from the liquid surface by a pulsed, tunable probe laser beam. The LIF emission was collected by a system of lenses and detected by a photomultiplier tube (PMT).

**Figure 1:**
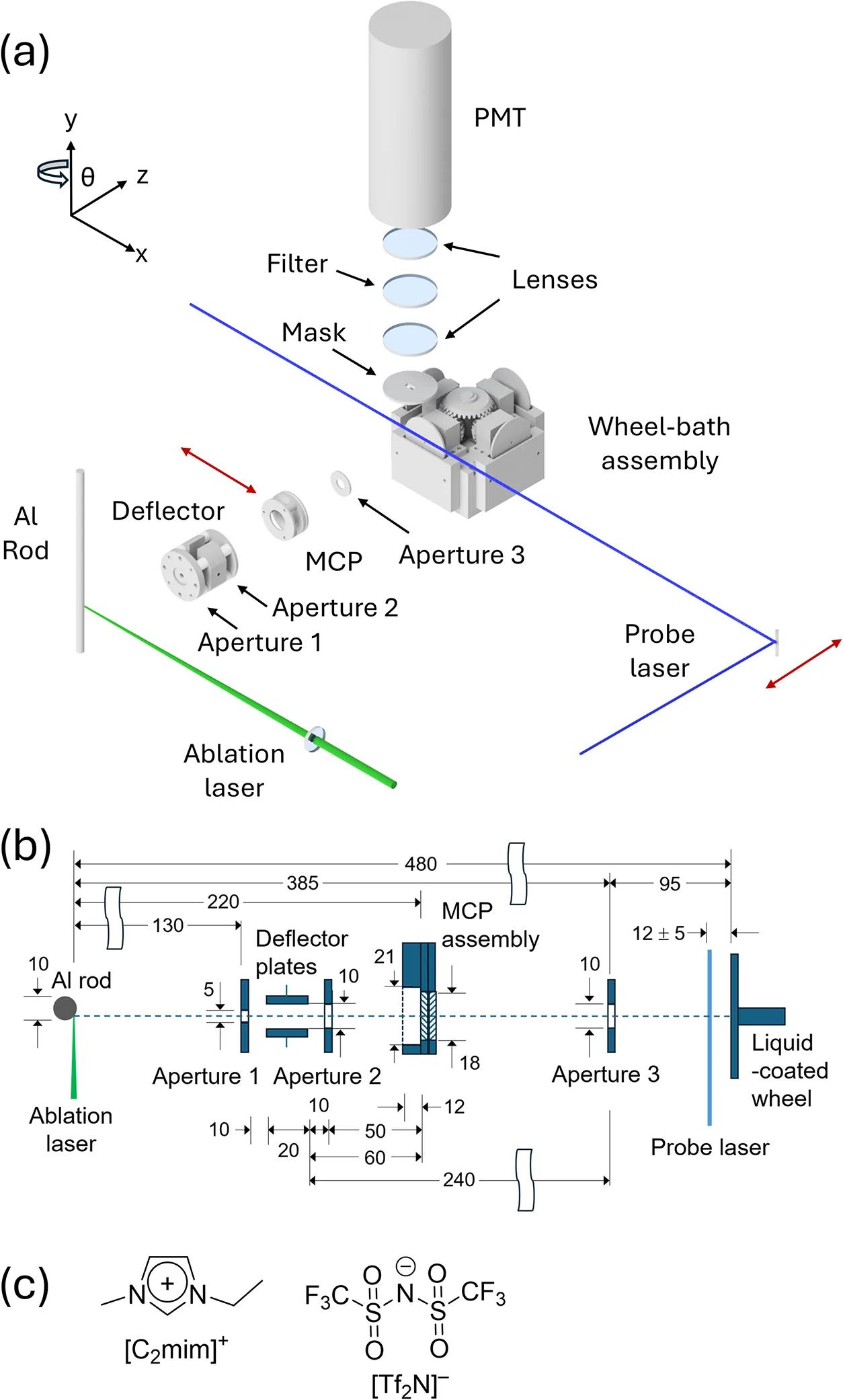
(a) Schematic diagram of the experiment setup, with definition of the axis system. Notable changes from previous iterations^14,15^ include a set of deflector plates, a retractable MCP detector used to detect cations, and a translatable mirror in the probe laser beamline which allows the surface-to-probe distance to be varied. The first two apertures are positioned directly before and after the deflector, respectively. (b) Schematic plan view of the key elements showing distances and aperture dimensions (all in mm). Not to scale; note the breaks indicated in the longer distances. (c) Chemical structure of the 1-ethyl-3-methyl-imidazolium cation, [C_2_mim]^+^, and the fluorine-containing bis­(trifluoromethylsulfonyl)­imide anion, [Tf_2_N]^−^, in the IL studied here.

Three key changes were made to enable the new experiments here, also indicated in Figure 1a. First, a retractable commercial microchannel plate (MCP) detector assembly (Photonis USA Inc., 18 mm diameter active area, Chevron stack with metal anode) was introduced between the Al source and the liquid surface. This allowed the direct detection of positive ions (with the biases applied to the plates in this work) in the incident beam and the characterization of their KE distributions by TOF measurements. The ion current at the anode was recorded on an oscilloscope. The detector was attached to a flange-mounted plate which could be translated in and out of the incident beam using a linear manipulator. The front face of the MCP detector was positioned 220 mm from the point on the Al rod where ablation took place, with a grounded meshed front plate (open-aperture diameter = 21 mm) positioned 12 mm from the front of the detector.

The time zero of the TOF spectrum was determined using a fast photodiode to detect the ablation pulse at its entrance window to the chamber. This agreed with Q-switch delay settings and was corroborated by an optical signal, most likely short-wavelength emission from the plasma created in the ablation, picked up by the MCP detector. This transient pulse, which became more intense at higher ablation pulse energies, and the strong ringing at the anode that accompanied it, was screened out from the final TOF spectrum by applying a 4 MHz low-pass filter during the data analysis process.

Second, a pair of electrostatic deflector plates was mounted onto the first bulkhead, positioned between the source and the MCP detector, 10 mm beyond Aperture 1 (5 mm diameter) – see Figure 1b. The purpose of the deflector was 2-fold: to monitor the changes to the TOF spectrum as ions were deflected away from the MCP detector; and, when the MCP detector was retracted, to allow any corresponding changes to AlF production at the liquid surface resulting from deflecting ions away from Aperture 3 (10 mm diameter, 95 mm from the liquid surface) to be determined by LIF. The deflector plates were 20 mm long and separated by 10 mm. Aperture 2 (10 mm diameter), which acts as an exit ground plate, was positioned 10 mm beyond the end of the deflector plates. The deflector was orientated to deflect positive ions in the positive-*x* direction (see Figure 1a) off the central axis of the chamber (*i.e*. *z*-axis). The electric field strength was varied up to a maximum of 250 V cm^–1^ by applying equal but opposite voltages to the two plates.

Third, the final mirror directing the LIF probe laser beam into the target chamber was mounted on a fine-control translation stage. This allowed the perpendicular distance (along *z*) between the beam and the parallel liquid-coated surface of the wheel to be controlled. This was done primarily to determine the speed of the AlF product returning from the liquid surface by recording the AlF LIF profile at different probe-laser distances. Previous studies had used a set distance of nominally 10 mm;^14,15^ more precise measurements here established this to be closer to 12 mm, taking account of the offset due to the Brewster-angle entrance window. Measurements here spanned 7 to 17 mm, set precisely using two translatable irises mounted on the chamber outside the entrance and exit windows, respectively.

Other aspects of the experiment were similar to those in our most-recent previous work.^15^ The ablation pulse energy (second harmonic (532 nm) output from a Continuum Surelite I-10 Nd:YAG laser; ∼5 ns pulse) was controlled using a λ/2 waveplate relative to a fixed linear polarizer. The light was focused onto the cylindrical (diameter = 10 mm) Al rod by a 300 mm focal length lens. The rod was rotated after every fifth laser shot, before it was vertically translated after a near-full rotation (355°), to achieve consistency in Al species production. The ablation point was 480 mm from the liquid surface. The same liquid-wheel assembly was used, but only one wheel was required for the single IL investigated here; for LIF measurements of Al in the incident beam, the assembly was rotated to a position from which the wheel had been removed, to eliminate the possibility of detecting Al scattered from the liquid surface.

The LIF probe laser light was generated by frequency doubling the fundamental output of a Nd:YAG-laser pumped dye laser. The AlF LIF signal was generated on the A^1^Π-X^1^Σ^+^ transition around 227 nm. Typical pulse energies were 0.25–0.50 μJ in a ∼5 ns pulse. LIF was detected along the *y*-direction by a system of lenses and PMT. AlF excitation spectra were obtained by recording the LIF intensity while scanning the probe wavelength. AlF appearance profiles were generally measured on the strongest feature, the Q-branch bandhead of the (0,0) band at 227.4805 nm.^14,15,70^ They were normally averaged over 50 laser shots for each delay between ablation and probe pulses, with a step size of 2 μs. The delays were chosen at random within a predefined range to minimize the effects of any systematic drift caused by source production and laser instabilities. Multiple (typically five) individual profiles were recorded for each set of conditions. Each of these sets of measurements was repeated several times on different days to ensure reproducibility.

Ground-state Al atoms were probed by LIF on the ^2^D_5/2_ ← ^2^P_3/2_^o^ transition at 226.910 nm.^14,71^ TOF profiles were recorded using similar averaging procedures to the AlF appearance profiles, including at a range of surface-to-probe distances as noted above.

Independent measurements were also made using a separate source-block ablation instrument to determine which charged species were produced in significant yield from an Al target under similar ablation fluences (*i.e*. comparable pulse energies and with a lens of the same focal length (300 mm)). They were done under conditions where the initial KE from ablation is dissipated in a carrier gas (He). This decouples TOF measurements from the effect of this energy, making them an unambiguous determination of mass-to-charge (*m*/*z*) ratio alone. The source-block ablation assembly itself has been described in previous publications,^7,72–74^ and details of this instrument and the experiments done for this work are provided in the SI (Section S1, Figure S1).

## Materials

The Al rod used in the RAS-LIF experiments was made of HE30TF/6082 grade aluminum metal with a stated purity of 95–98%. The Al and Li:Al alloy disc targets used in the source-block ablation setup (see SI, Section S1, Figure S1) were punched out of thin foils (Goodfellow) and attached to a metal mounting disc using adhesive.

The chemical structure of the single IL used in the scattering experiments, [C_2_mim]­[Tf_2_N], is shown in Figure 1c. Details of its synthesis have been reported previously.^14^ This IL was chosen both because it is in widespread use and because it gave a strong AlF LIF signal in previous studies, consistent with the short alkyl chain on the cation and the high fluorine content on the counteranion.^15^ The IL was degassed in a separate purpose-built vacuum setup at a pressure below 10^–6^ mbar for 3 h before being transferred to the main experimental vacuum chamber.

## Results

### Speciation of Ions Produced by Ablation

Figure 2 shows the mass spectra of the laser ablation products from the Al and Li:Al alloy targets obtained in the source-block ablation apparatus. The ablation pulse energy was 3–4 mJ. Both spectra contain a dominant peak centered at *m*/*z* = 27 amu that corresponds to Al^+^. The peaks are slightly asymmetric, with a tail to higher *m*/*z* values. The origin of this effect is well understood to be due to the finite dimensions of the in-line TOF detection employed. When the length of the ion packet exceeds that of the Wiley–McLaren repeller-extractor region (2 cm), then those ions that are excluded and only accelerated within the extractor-ground region contribute to the low-energy (apparently higher mass) tail. In both panels, there are no peaks that correspond to AlO^+^ or AlOH^+^ (*m*/*z* = 42.98 and 43.99 amu, respectively), which implies that there is no significant ion production from any surface oxide or adsorbed water. There is also no evidence of any aluminum cationic dimers, Al_2_^+^ (*m*/*z* = 53.96 amu), or multiply charged Al ions, Al^*n*+^. In addition, there are also readily detectable Li^+^ signals from ablating the Li:Al alloy, with intensities that match the isotopic distribution expected for ^6^Li and ^7^Li (7.5 and 92.5%, respectively).^75^ There is no evidence of LiO^+^ or LiOH^+^ at 22.94 and 23.95 amu, respectively, consistent with the lack of oxygen-containing Al species. The observation of the Li^+^ signals assisted in calibrating the mass spectra and confirms that the instrument has sensitivity to lower *m*/*z*, necessary to exclude significant production of Al^*n*+^ species from either target. No Li:Al targets were used in the RAS experiments that follow.

**Figure 2:**
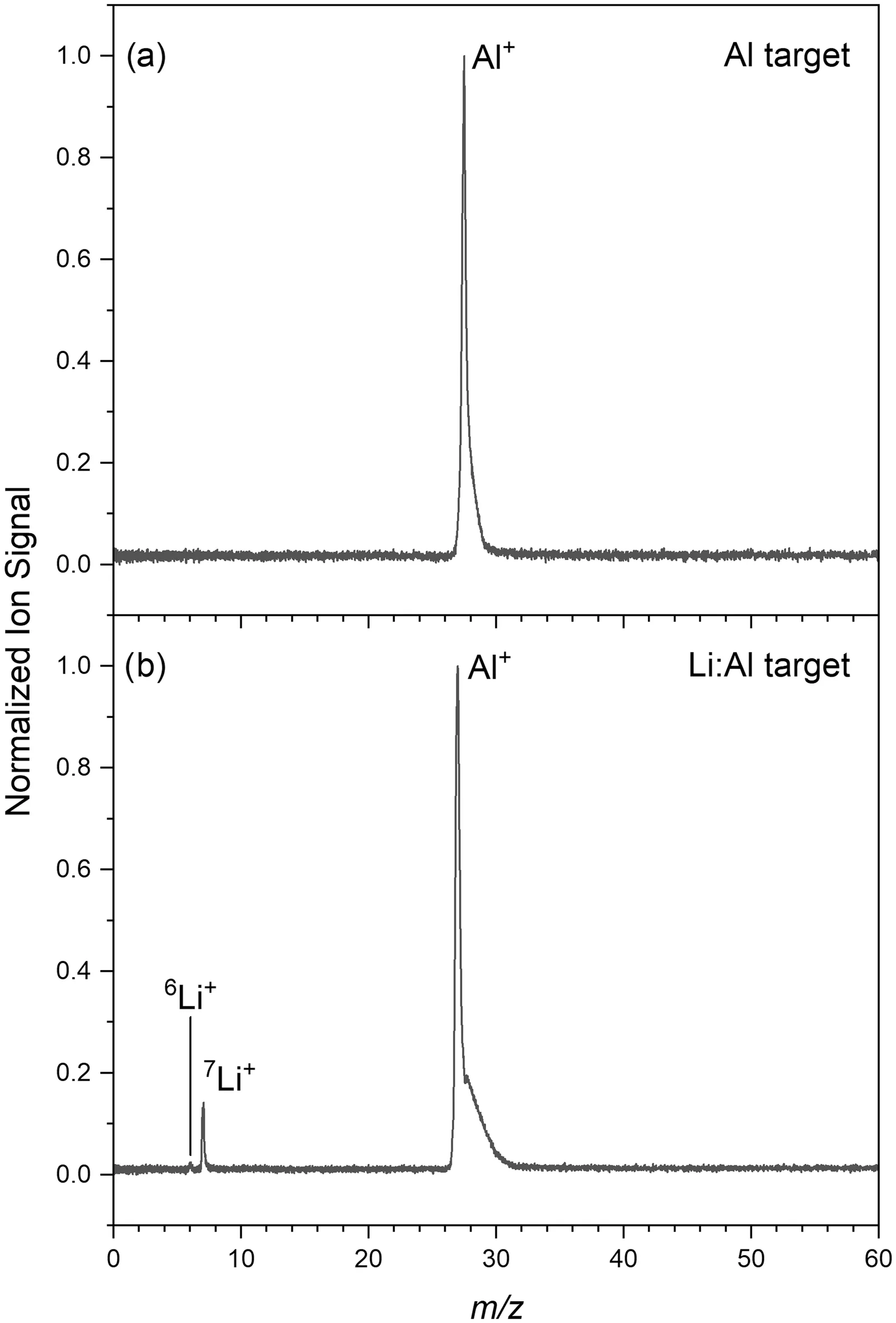
TOF mass spectra of products from laser ablation of Al (panel a) and Li:Al alloy (panel b) disc targets. For the Al disc, only Al^+^ is observed, with no detectable AlO^+^, AlOH^+^, Al^*n*+^ (*n* ≥ 2), or Al_2_^+^ signals. For the Li:Al disc, both the ^6^Li^+^ and ^7^Li^+^ isotopes are observed, confirming the mass calibration and sensitivity to ions with lower *m*/*z*.

### TOF Measurements of Al^+^ Ions Produced by Ablation

TOF profiles were recorded for the ionic species using the MCP detector in the RAS-LIF apparatus over a range of ablation laser pulse energies. Figure 3 shows the TOF profiles (ion flux as a function of time after the ablation pulse) recorded for 1, 2, 8, and 30 mJ pulse^–1^. Based on our previous estimate of an ablation laser spot area of 7.07 × 10^–4^ cm^2^,^14^ these correspond to fluences of 1.4, 2.8, 11, and 42 J cm^–2^, respectively. The profiles have been normalized relative to the peak signal in the 30 mJ pulse^–1^ trace.

**Figure 3:**
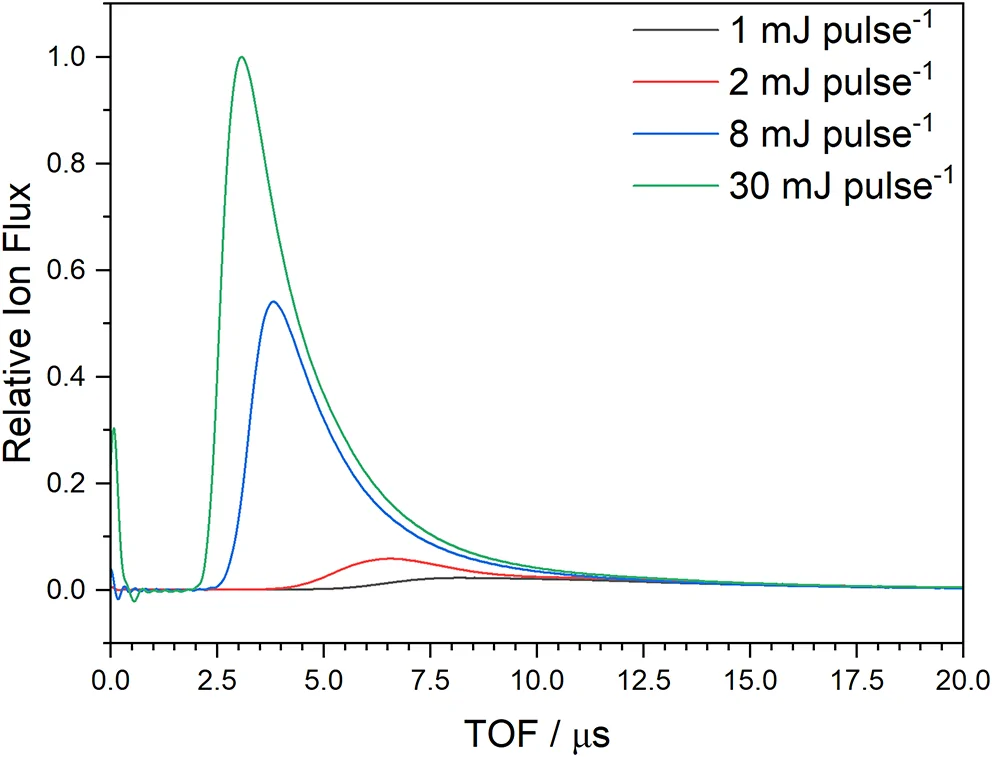
TOF profiles for Al^+^ for a range of ablation laser pulse energies (1, 2, 8, and 30 mJ), recorded on an MCP detector situated 220 mm away from the point of ablation. Fluxes are normalized relative to the peak of the trace for 30 mJ pulse^–1^. The spike at early times, most-visible at the highest pulse energy, is due to detection of optical emission coincident with the ablation pulse. Its intensity has been reduced by postapplication of a low-pass (4 MHz) digital filter, which also suppresses associated artificial ringing.

The peak position in Figure 3 shifts to earlier times with increasing ablation energy, from ∼8.2 μs at 1 mJ pulse^–1^ to ∼3.1 μs at 30 mJ pulse^–1^. The obvious interpretation is that the KE of a single ionic species is increasing with ablation energy; we assume this to be Al^+^ from the evidence already presented in Figure 2, supported by the absence of any significant sharp multimodality in the data in Figure 3. As indicated above, the Al^+^ species are detected 220 mm from the point of ablation. Ignoring a minor correction for acceleration of the ions across the final 12 mm between the grounded grid and the MCP detector face, the peak arrival time at the highest pulse energy gives an indicative Al^+^ speed in excess of 70,000 m s^–1^, which corresponds to a KE of ∼69,000 kJ mol^–1^ (or ∼720 eV). The TOF profiles in Figure 3 have been transformed, including the usual Jacobian factors, into full speed and energy distributions; these are provided in the SI (Section S2, Figure S2). The energies of the Al^+^ at the peak of the TOF distribution, and the calculated average energy, are listed in Table 1.

**Table 1:** Kinetic Energies of the Al^+^ and Al Species at Different Ablation Pulse Energies^a^

pulse energy/mJ pulse^–1^	Al^+^ energy at peak/eV	average Al^+^ energy/eV	Al energy at peak/eV	average Al energy/eV
1	100	76		
2	158	111	11	17
4			16	18
8	466	316	16	19
30	721	456	16	17

a The energies were derived from the Al^+^ MCP detector measurements (see Figure 3), and the Al LIF TOF profiles (see Figure 5), respectively. Peak values correspond to the KE at the peak of the TOF profile. For Al, the average energy was calculated using the earlier peak (delay between 20 and 100 μs) only, for the reasons explained in the text. All values are rounded to the nearest integer.

As is obvious from Figure 3, the magnitude of the ion signal also increases strongly with the ablation pulse energy. This is also shown in Figure 4 (also see SI, Section S3, Table S1), which includes Al^+^ fluxes integrated across the full TOF profile. For reasons of comparison with other integrated quantities to follow, the Al^+^ integrated fluxes are normalized at an ablation energy of 8 mJ pulse^–1^ in Figure 4. They increase approximately linearly with ablation-pulse energy initially but begin to plateau at higher pulse energies.

**Figure 4:**
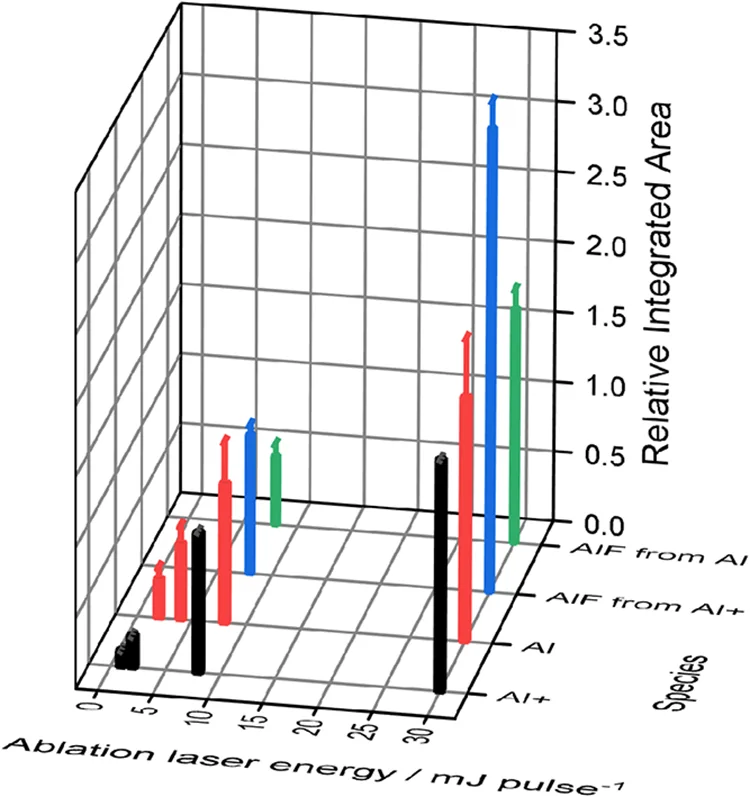
Relative integrated fluxes toward the surface (Al^+^ and Al) and integral AlF product signals as a function of ablation laser energy. Al fluxes were produced by transforming the Al LIF number densities; the integrated area only includes the faster peak (between 20 and 100 μs). The AlF data are derived from LIF appearance profiles (*i.e*. are integral number densities rather than fluxes, but see text). The relative detection sensitivities for different species are unknown, so the yields of Al, Al^+^ and AlF from Al^+^ have been normalized at 8 mJ pulse^–1^. The ratio of AlF yields from Al^+^ and Al is as measured directly. The ratio of AlF signals at 8 and 30 mJ pulse^–1^ has been fixed at the value of 0.36 determined accurately previously.^15^ Error bars represent 1σ standard errors in the mean of repeated measurements.

The effect of the deflector on the Al^+^ TOF profiles was also investigated; the primary aim was to confirm that effectively all charged species could be deflected out of the beam to prevent them reaching the IL surface and hence determine any contribution they make to AlF production, as follows below. If ions can be deflected off the MCP detector they would certainly not reach the IL surface situated at a significantly larger distance beyond Aperture 3 – see Figure 1b. Figure 5 shows a stacked plot of the TOF profiles at the MCP detector for the same sequence of ablation pulse energies as in Figure 3, for a range of deflector field strengths (0 to 250 V cm^–1^, 50 V cm^–1^ step size; the black traces in Figure 5 correspond to those in Figure 3). For the lowest ablation pulse energy (1 mJ pulse^–1^), the signal is reduced significantly by applying a field strength of 50 V cm^–1^ and is removed completely by 100 V cm^–1^. As anticipated, only the faster ions at earlier times survive as the field strength is increased and this pattern is repeated as the ablation pulse energy is increased, with progressively higher field strengths being needed to remove the ions at earlier times. At 30 mJ pulse^–1^, the highest field strength applied of 250 V cm^–1^ is not quite sufficient to deflect all the ions away from the detector, with a small but distinguishable residual signal at ∼2.4 μs. This would equate to an Al^+^ speed of more than 90,000 m s^–1^ and a KE of ∼1.2 keV, consistent with the speed and energy distributions shown for 30 mJ pulse^–1^ (see SI, Figure S2), where a notable proportion of the distribution extends beyond KEs of 1 keV.

**Figure 5:**
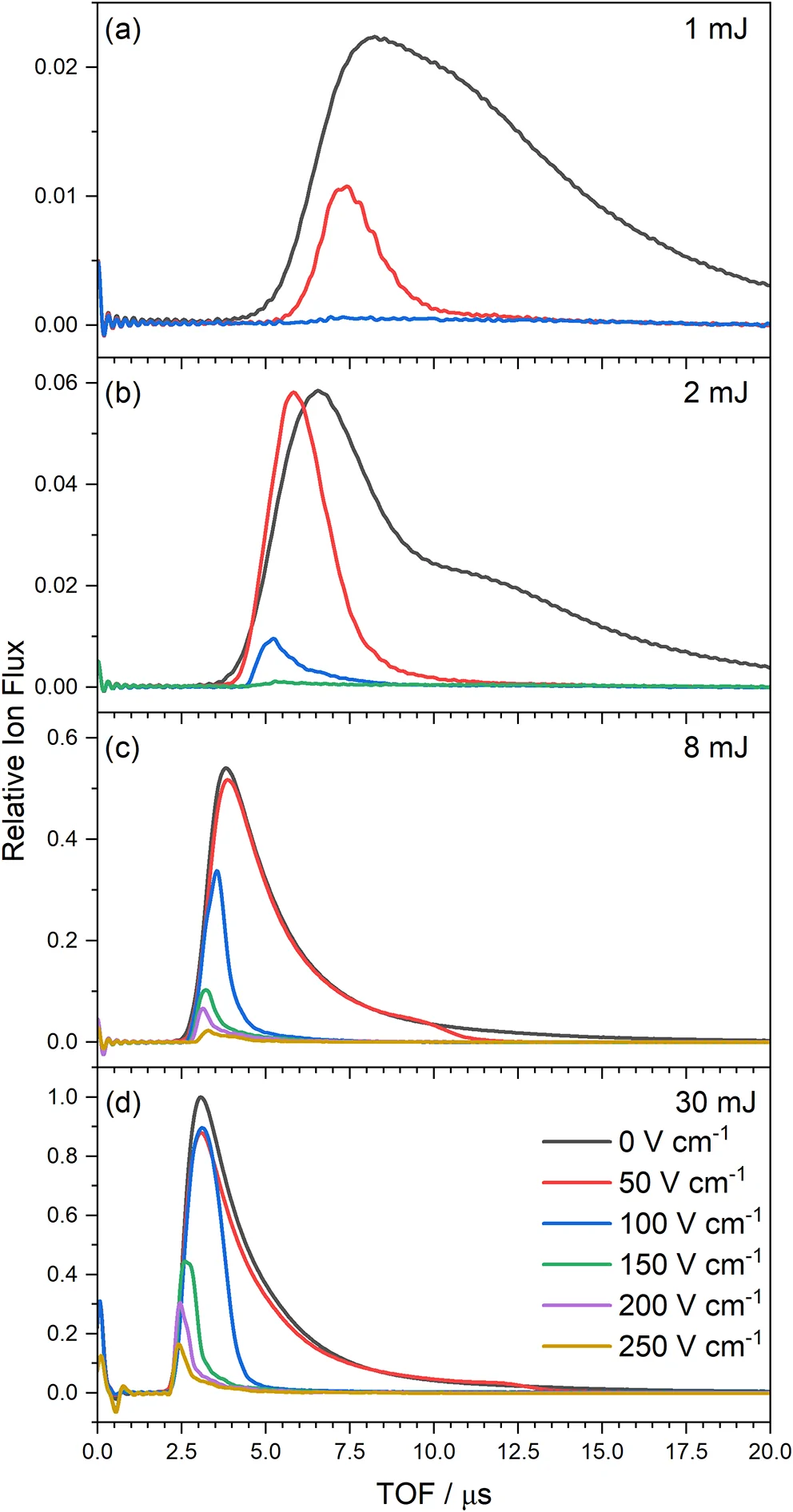
Effect of the deflector on the TOF profiles for Al^+^ across a range of ablation laser pulse energies (a) 1, (b) 2, (c) 8, and (d) 30 mJ, recorded on an MCP detector situated 220 mm from the point of ablation. The electric field strengths between the deflector plates are as indicated in panel (d). The data in all panels are normalized relative to those at the highest pulse energy (*i.e*. 30 mJ pulse^–1^) with no potential applied to the deflector (field strength = 0 V cm^–1^, black trace in panel d); note the variations in the vertical-axis scale.

These deflector studies had two secondary benefits. They exclude the possibility that fast neutral species might have been responsible for a significant component of the signal produced by the MCP detector. They also provide some independent corroboration of the KEs of the ions derived from their times of flight. As shown in the SI (Section S4, Figure S5), the fields required to deflect the ions away from the MCP detector are at least approximately consistent with the KEs of the ions, based on their TOF, and the known geometry of the apparatus. However, there is some survival of ions at later times than would be predicted, which is more significant at higher ablation fluences. There are a number of possible explanations, which we have not pursued in detail but are described in the SI (Section S4). This does not materially affect any of the conclusions reached in this work.

### LIF TOF Measurements of Al Atoms Produced by Ablation

Figure 6 shows the TOF number-density profiles for the neutral Al atoms for a range of ablation pulse energies from 2 to 30 mJ pulse^–1^. The LIF intensity in each panel is normalized relative to the peak of the signal in the 30 mJ pulse^–1^ trace. These measurements were made with the LIF probe beam 468 mm from the point of ablation (which would correspond, for context below, to a liquid surface-to-probe distance of 12 mm if the wheel had been in place (see Figure 1b); however, as noted above, it was removed for these measurements).

**Figure 6:**
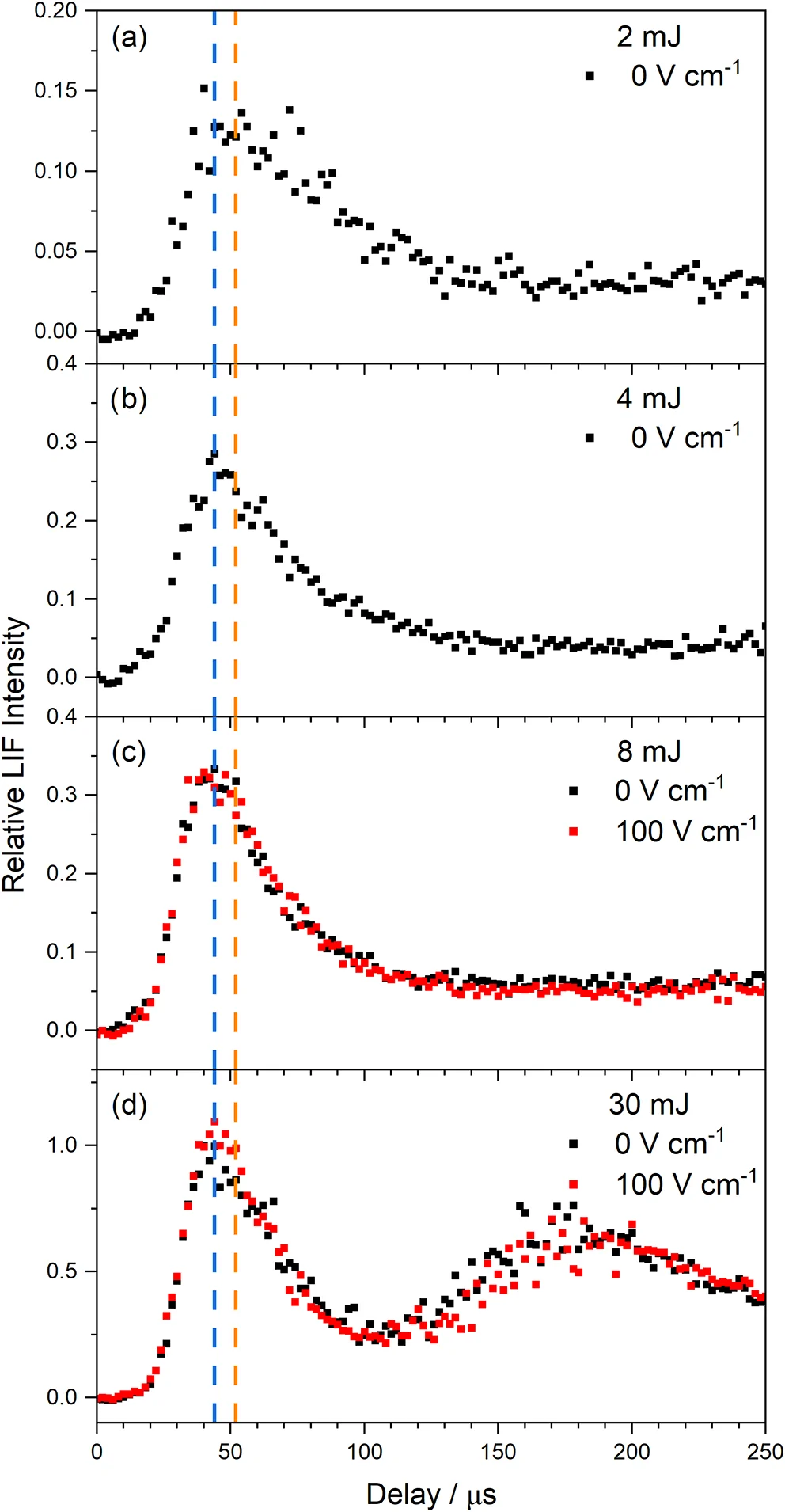
LIF TOF profiles of Al atoms using ablation pulse energies of (a) 2, (b) 4, (c) 8, and (d) 30 mJ. For 8 and 30 mJ, the profiles were recorded with the deflector turned off (0 V cm^–1^, black squares) and on (100 V cm^–1^, red squares). The orange and blue dashed lines mark the estimated peak positions in the 2 and 30 mJ pulse^–1^ profiles at 52 and 44 μs, respectively.

The dominant peak at 2 mJ pulse^–1^ is at ∼52 μs, corresponding to a speed of ∼9000 m s^–1^, or a KE of 1035 kJ mol^–1^ (11 eV). For all three higher pulse energies (4, 8, and 30 mJ pulse^–1^), the dominant peak is at an almost constant and slightly earlier time (∼44 μs), corresponding to a speed of ∼10,600 m s^–1^ and KE of 1520 kJ mol^–1^ (16 eV). Unlike with Al^+^ above, this implies that the additional pulse energy is not efficiently coupled into Al translation. Density-flux transformed versions of the Al TOF profiles are given in the SI (Section S2, Figure S3). The peak and average KEs of the Al are compared with those of the Al^+^ in Table 1; the averages were derived by integrating over the region in the KE flux distributions corresponding to the earlier peaks in the TOF distributions. There is around an order of magnitude difference in KE between Al^+^ and Al at lower fluences, with the ratio increasing in favor of Al^+^ at higher fluences.

The data in Figure 6a–c also show a significant broad plateau extending out to longer times; a similar feature was noticed in our earlier work using lower fluences.^14^ At the highest ablation energy in Figure 6d, the dominant early peak is still present, but the signal at later times has now developed into a clear secondary maximum at ∼180 μs; this corresponds to a speed of ∼2700 m s^–1^ or a KE of ∼100 kJ mol^–1^ (1.0 eV).

Neither the earlier nor later peaks are affected significantly by the application of the deflector voltage (red points, Figure 6c,d). This is, of course, as expected if the neutral Al atoms being detected have flown directly from the ablation source. It excludes the possibility that some of the Al atoms, potentially more likely to have been those in the later peak, might have been produced by Al^+^ colliding with and being neutralized at surfaces in the apparatus downstream from the deflector region. Moreover, we have also confirmed that the positions of both peaks are barely affected, within the signal-to-noise, when the location of the LIF probe beam is moved by ±5 mm in the direction of travel of the Al beam (see SI, Section S5, Figure S5). This is also exactly as expected for these changes being a small fraction of the distance traveled (468 mm) from the point of ablation. This reinforces the elimination of Al^+^ neutralization and, additionally, of inelastic scattering of Al atoms themselves from a surface in the apparatus closer to the LIF probe point as the source of the Al atoms at later times. We conclude that all the evidence points to the later peak being a genuine slower component of Al atoms produced in the ablation.

As the pulse energy increases, the relative intensity of the most intense peak obviously increases (note the changes of scale in Figure 6). Quantitative relative fluxes of the faster Al peak as a function ablation laser pulse energy were determined from the density-flux transformed versions of the profiles – as noted above, these are given in the SI (Section S2, Figure S3). The results are included in Figure 4, where they follow a similar pattern to the Al^+^ integral fluxes, increasing approximately linearly at low fluences but less so at higher fluences.

### LIF Measurements of AlF Appearance Profiles from Reaction at the IL Surface

LIF appearance profiles for the AlF produced from reactive scattering from the [C_2_mim]­[Tf_2_N] surface were recorded using two different pulse energies (8 and 30 mJ pulse^–1^), as well as a range of deflector field strengths (0 to 100 V cm^–1^; 20 V cm^–1^ step size), as is shown in Figure 7. The profiles were recorded with a surface-to-probe distance of 12 mm. Low deflector field strengths (20 V cm^–1^, red squares) have little appreciable effect on the profiles at either ablation pulse energy. As the field strength is increased further, the AlF LIF signal decreases; this corresponds to the contribution from reaction of Al^+^ being reduced as the slower Al^+^ ions fail to pass through Aperture 3. A shoulder feature becomes more pronounced at earlier delays, assigned to the contribution from the faster Al^+^. The AlF LIF signal continues to decrease until around 80 V cm^–1^, with only a small shoulder for the 30 mJ pulse^–1^ profile. At 100 V cm^–1^ (or beyond, in other similar measurements up to 200 V cm^–1^ – see the SI (Section S4, Figure S6)), the remaining AlF is effectively being produced only by the neutral Al atoms.

**Figure 7:**
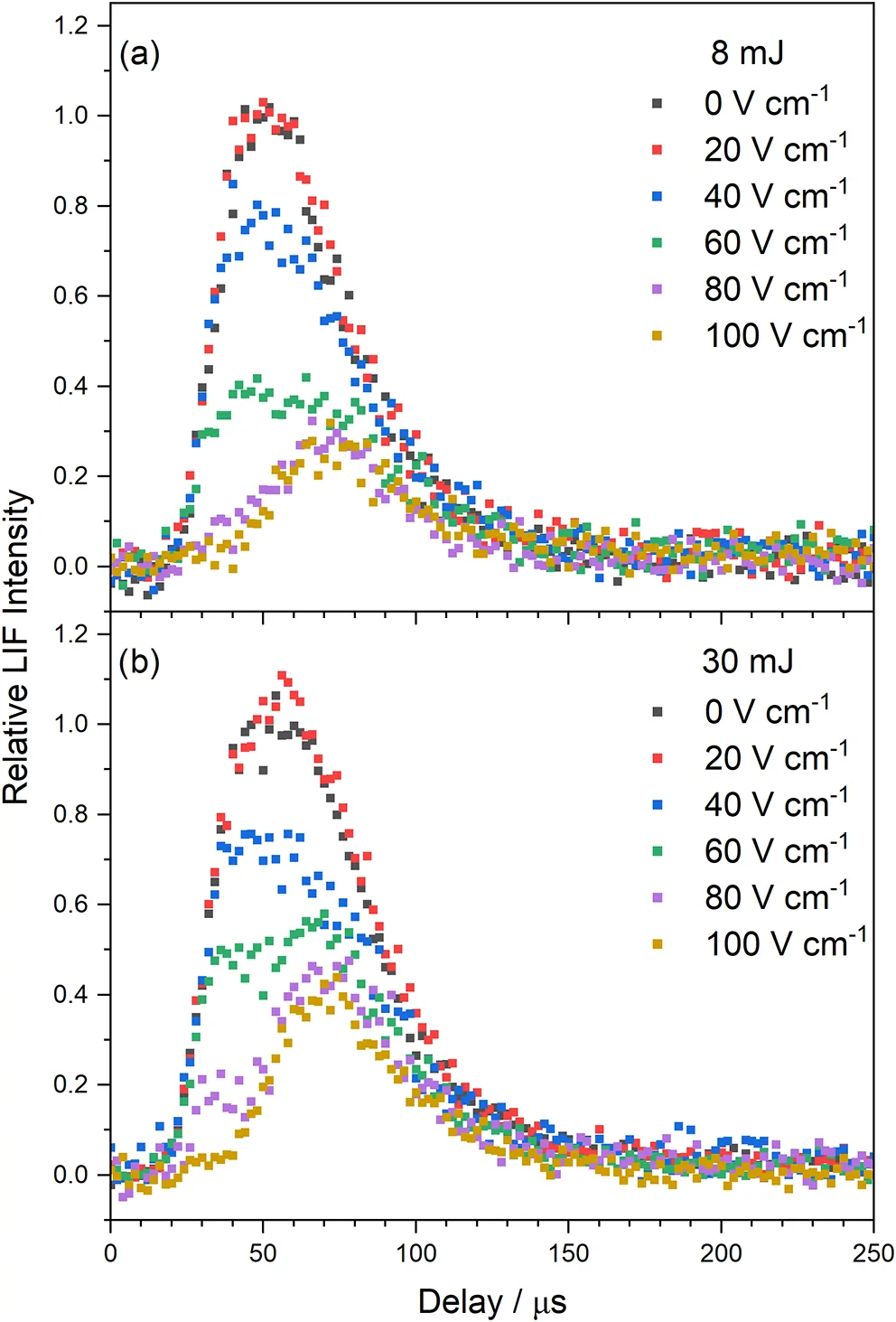
LIF appearance profiles of AlF produced via reactive scattering of Al species on the [C_2_mim]­[Tf_2_N] liquid surface. The profiles were recorded with an ablation pulse energy of (a) 8 and (b) 30 mJ, and across a range of deflector electric field strengths (0–100 V cm^–1^), as shown. The data in each panel are normalized relative to those with an electric field strength of 0 V cm^–1^ (black squares).

The contributions to AlF production from the two projectiles were derived by taking the difference between the deflector-off and deflector-fully on profiles (for Al^+^) and the deflector-fully on profile only (for Al). This separation is illustrated for the 30 mJ pulse^–1^ data in Figure 8a. The relative yields were obtained by integrating the two respective features at each ablation fluence. While strictly these are integral AlF number densities, it is shown below that the speed distributions from the two projectiles are very similar (both close to thermal), meaning that the number-density ratios will be very close to flux ratios. The results are included in Figure 4 (see SI, Section S3, Table S1 for numerical values), where the ratios of AlF produced by Al^+^ and Al are almost the same (very close to 2:1) at both fluences. The ratio of signals between 8 and 30 mJ pulse^–1^ was set at the value (0.36) that we had determined accurately in our previous work.^15^

**Figure 8:**
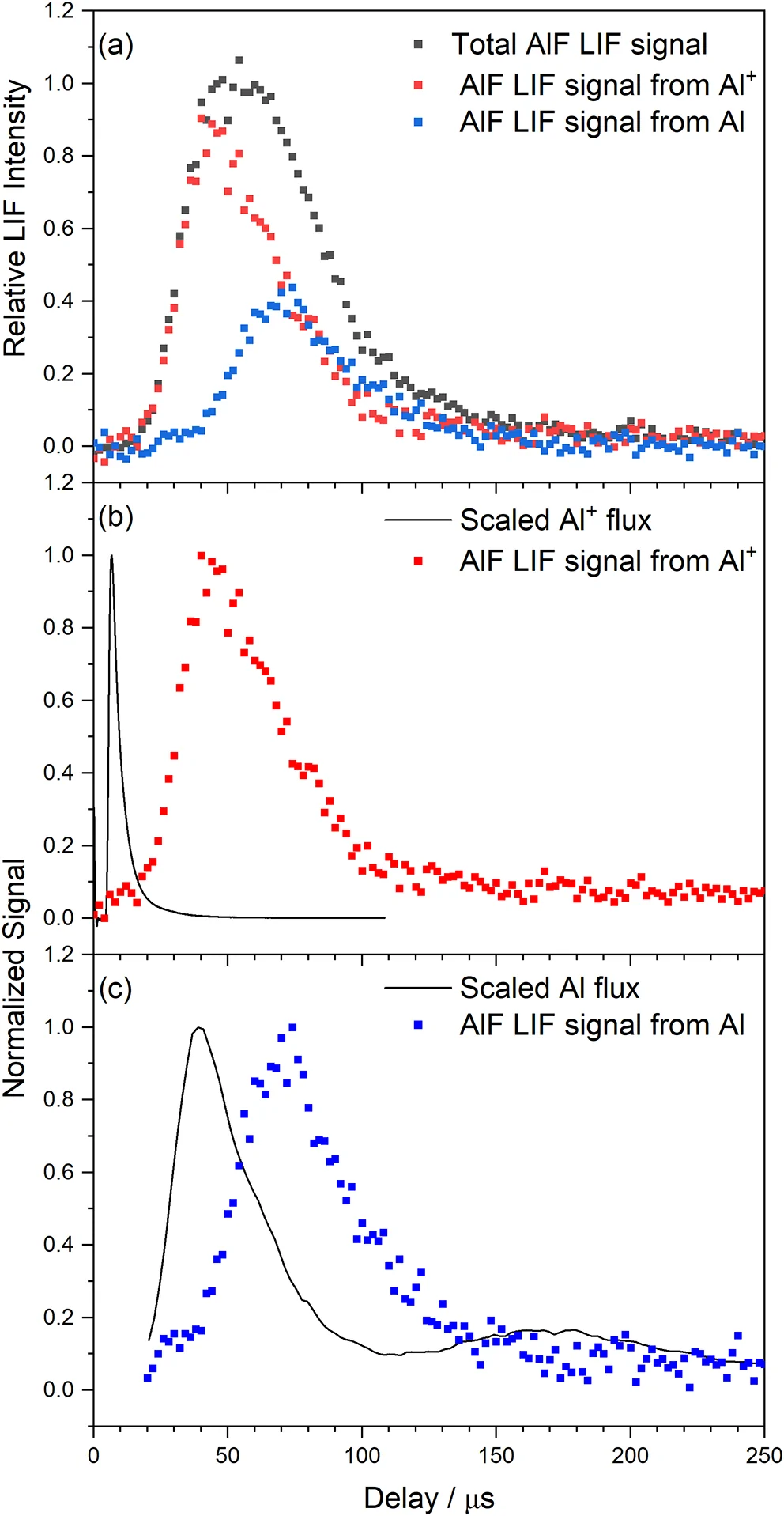
Deconvolution of AlF signal recorded at 30 mJ pulse^–1^. (a) Total AlF signal (deflector off: black squares); AlF from Al^+^ (deflector off – deflector on: red squares); AlF from Al (deflector on: blue squares). (b) Projected incident flux at the IL surface of Al^+^ (black line); peak-normalized AlF LIF from Al^+^ (red squares). (c) Projected incident flux at the IL surface of Al (black line); peak-normalized AlF LIF from Al (blue squares); data are only presented from 20 μs onward because of numerical instabilities at earlier delays.

It is clear from Figure 7 or Figure 8a that the Al^+^-induced contribution to the AlF signal peaks significantly earlier than that from Al. Qualitatively, this is consistent with the Al^+^ ions arriving earlier at the surface than the Al atoms, as implied by proper interpretation of Figures 3 and 6 (noting the different distances at which measurements were made). Figure 8b,c have been constructed to illustrate this more quantitively and eliminate trivial differences between Figures 3 and 6 due to the Al^+^ and Al TOF profiles being measured at different distances from the point of ablation. The projected incident flux profiles at the position of the IL surface are shown (solid black lines) in Figure 8b for Al^+^ and Figure 8c for Al, respectively. For Al^+^, this was done simply by scaling the time axis by the ratio of distances from the ablation point to the IL surface and MCP detector, respectively (*i.e*. 480 mm/220 mm). For Al, the measured LIF TOF profile was first transformed to flux. A minor correction was then made for the slightly larger distance to the IL surface than to the LIF measurement point (*i.e*. 480 mm/468 mm).

These incident profiles are coplotted with their associated contributions to the AlF LIF appearance profiles (red or blue points in Figure 8b,c, respectively). As is apparent, the resulting differences between the peak of the incident projectiles and the outgoing AlF profiles are very similar; ∼33 μs for the Al atom, and ∼37 μs for the Al^+^ cation. This implies that the AlF has similar speeds, independent of whether it is produced from Al^+^ or Al; this was the basis of the claim above that the relative fluxes are similar to the relative number densities. Note also in passing that the slower Al peak at around 180 μs in Figure 8c does not produce any obvious secondary wave of AlF, whose significance is considered below.

It is difficult to make a reliable estimate of the absolute AlF speeds based on this single-point measurement at a relatively short distance between the IL surface and the LIF probe beam, as the finite sizes of the dosed area and the length of the LIF probe volume result in a non-negligible spread in the distances traveled between surface and probe. This is compounded by the significant spread in arrival times of the incident projectiles, especially for Al atoms. The most-probable speed was evaluated more robustly by varying the distance between the IL surface and the LIF probe beam, where these effects have much less influence on relative peak positions. Figure 9a shows the AlF LIF appearance profile recorded at three different surface-to-probe distances (black = 7 mm, red = 12 mm, blue = 17 mm). The ablation pulse energy was 30 mJ; the deflector was turned off. As is apparent, there is the expected notable shift to later delays as the distance is increased.

**Figure 9:**
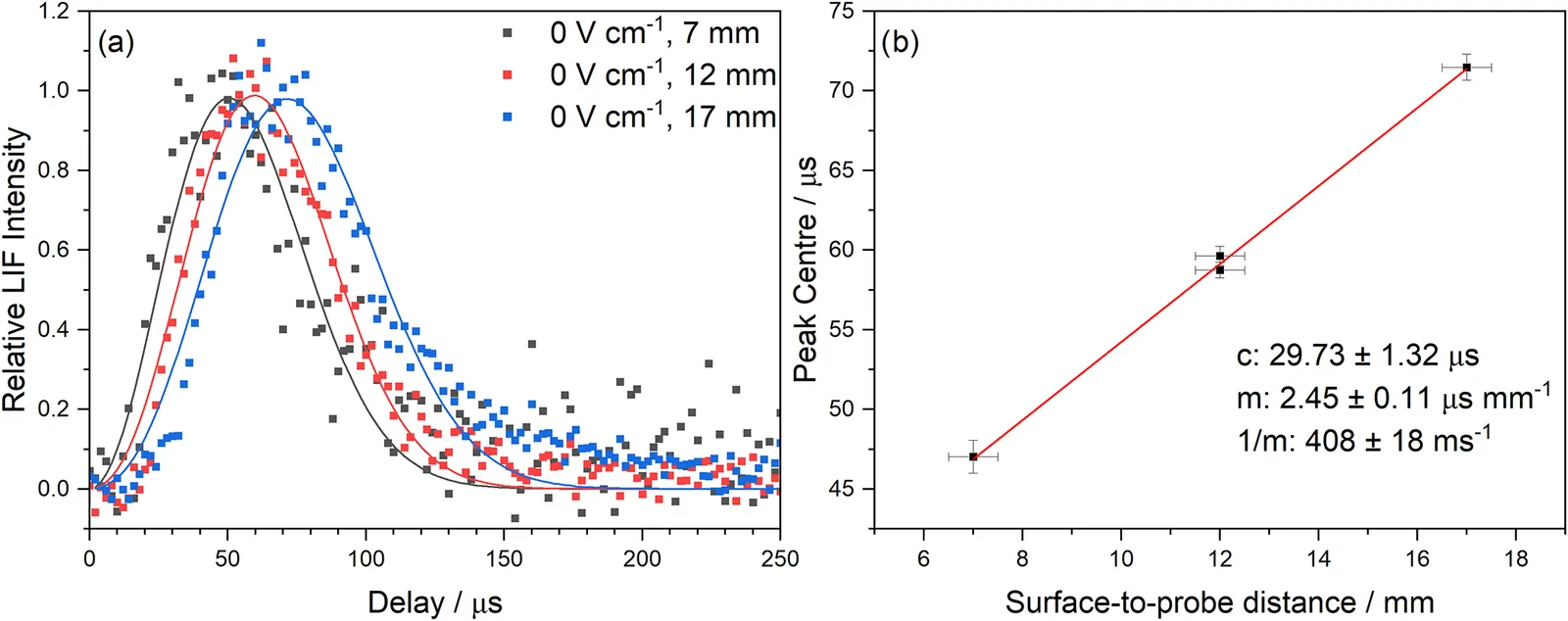
(a) AlF appearance profiles at different surface-to-probe distances. Squares represent the raw data, and the line is a phenomenological fit used to determine the peak position. (b) Peak positions as a function of surface-to-probe distance. The linear fit has a slope of 2.45 ± 0.11 μs mm^–1^, corresponding to a speed of 408 ± 18 m s^–1^ (1σ errors).

Given the limited signal-to-noise, unbiased estimates of the peak positions were obtained objectively by fitting an arbitrary empirical function of suitable form (see SI, Section S6, for details); the fits are included in Figure 9. These peak positions are plotted as a function of the surface-to-probe distance in Figure 9b; a linear fit gives an inverse slope of 408 ± 18 m s^–1^ (1σ uncertainty). We interpret this value as the speed at which the peak number density of the wave of AlF propagates away from the IL surface. For comparison, this can be predicted analytically for an assumed (unnormalized) Maxwell–Boltzmann number-density distribution of speeds, *v*, for a molecule of mass, *m*, at temperature, *T*:

1 $$P\left(v\right)dv\propto v^{2}\;\exp\left(\frac{-mv^{2}}{2\mathrm{kT}}\right)dv$$

where *k* is the Boltzmann constant. This can be transformed straightforwardly, including the usual Jacobian factors, to a TOF number-density profile at a distance, *z*:^14^

2 $$P_{t}\left(t\right)dt\propto \frac{z^{3}}{{\left(t-t_{0}\right)}^{4}}\exp\left(\frac{-mz^{2}}{2\mathrm{kT}{\left(t-t_{0}\right)}^{2}}\right)dt$$

where *t*_0_ is the time at which the AlF is created at the surface. This function peaks at

3 $$t_{\max}={t_{0}+\left(\frac{m}{4\mathrm{kT}}\right)}^{1/2}z$$

from which the apparent speed at which the peak of the wave propagates is

4 $$1/\left(\frac{dt_{\max}}{dz}\right)={\left(\frac{4\mathrm{kT}}{m}\right)}^{1/2}$$

Note that this differs by a factor of √2 from the most-probable speed of a Maxwell–Boltzmann number-density distribution.

For an assumed AlF temperature matching that of the liquid (320 K), eq 4 gives a propagation speed of 481 ms^–1^, which is close to the value estimated from Figure 9b. Given the limited signal-to-noise and the approximation of treating the entire AlF LIF profile as emanating from a single point source with no intrinsic temporal spread, we regard this as convincing evidence that the AlF has a predominantly thermal-like distribution of speeds at a temperature similar to that of the liquid. Furthermore, as shown in the SI (Section S7, Figure S8), by assuming a Maxwell–Boltzmann distribution of AlF speeds it is possible to predict accurately, with no adjustable parameters, the distance dependence of the position of the rising edge of the AlF appearance profiles.

### LIF Excitation Spectra of AlF Produced in Reaction at the IL Surface

We have reported previously that the AlF also has a near-thermal rotational distribution, but with an indication of residual vibrational excitation.^14,15^ We have investigated this further here by examining correlations between the AlF LIF excitation spectrum and time during the appearance profile. The results are shown in Figure 10, for an ablation pulse energy of 30 mJ. Delays have been chosen to correspond to the rising edge (30 μs), peak (50 μs) and falling edge (80 μs) of the AlF appearance profile (see Figure 7). A simulation using the PGOPHER program is shown for comparison,^76–79^ in which 320 K rotational populations are assumed in all vibrational levels of the electronic ground state, but with indicative vibrational populations adjusted to approximately match those of the experimental spectrum, as specified in the caption to Figure 10. The Q-branch bandheads of the higher vibrational levels, up to at least the (4,4) band, are clearly visible in the rising-edge spectrum. However, they are much less prominent, if present at all, in the peak and falling-edge spectra, which are barely distinguishable within the signal-to-noise ratio.

**Figure 10:**
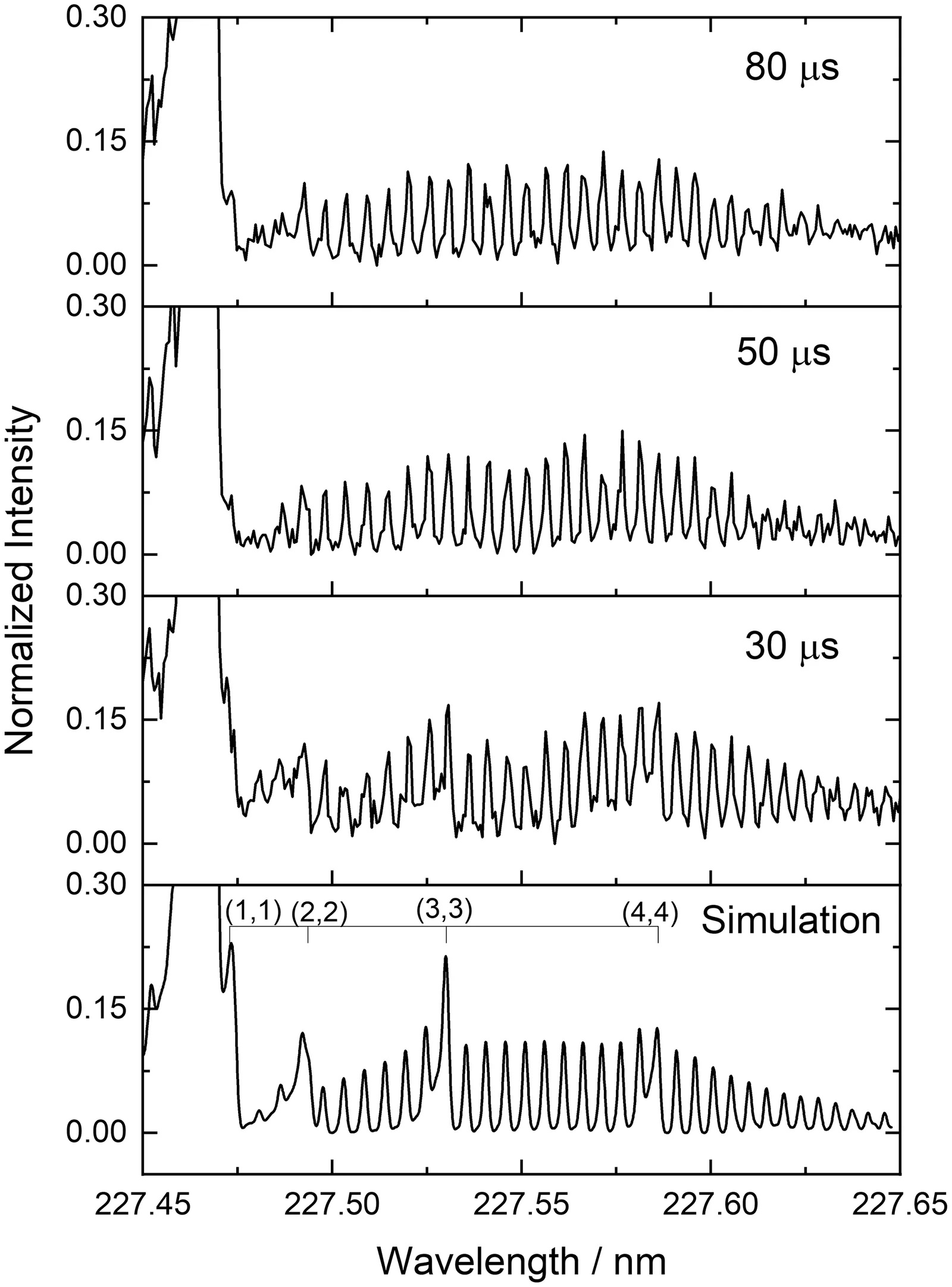
AlF LIF excitation spectra recorded at 30 mJ pulse^–1^ at delays corresponding to the rising edge (30 μs), peak (50 μs), and falling edge of the peak (80 μs) in the appearance profile (see Figure 7). The bottom panel is the PGOPHER simulation, including illustrative relative populations of 0.73:0.16:0.06:0.04:0.01 in vibrational levels *v* = 0–4, respectively; the Q-branch bandheads of the diagonal bands from vibrationally excited levels are indicated. The simulation has been convoluted with the estimated experimental instrumental line width, to aid visual comparison. All spectra are normalized to the peak of the Q-branch bandhead (off scale). A small correction has been applied to the experimental wavelength scale to bring it into register with the simulation.

## Discussion

### Laser-Ablation Process

We consider first what the observations reveal about the laser ablation of the Al target. Our previous work had confirmed the production of ground-state Al atoms through LIF detection, which we have confirmed and extended here.^14^ The mass spectra obtained with the source-block ablation instrument (Figure 2) indicate that the only cationic species produced in detectable yield is Al^+^. There are, however, some technical differences between the RAS-LIF and source-block experiments. The nominal fluence ranges overlap, but it is difficult to compare them directly for laser pulses with potentially different spatial and temporal profiles. The geometries are also slightly different; the source-block Al target is a flat foil, with the ablation beam directed along the surface normal, whereas the target in the RAS-LIF apparatus is a circular rod, irradiated at an angle to the local surface normal. Since the radius of curvature of the rod (5 mm) is large compared to the spot size (0.3 mm diameter), it is unlikely that the curvature itself is particularly significant. Note that the RAS-LIF geometry (compare Figure 1b) means that only the material ejected at a narrow subset of exit angles, centered around ∼45° to the local surface normal, reaches either the MCP detector (limited by Aperture 1; angular acceptance 2.2°) or IL surface (Aperture 3; 1.5°), whereas essentially all the ablated material is sampled in the source-block configuration. Perhaps most significantly, by design, the source-block experiment entrains the ablation plume in a carrier gas (He). It is possible, in principle, that collisional processes in the carrier gas affect the relative abundance of different Al^*n*+^ species, but we do not believe that is very likely based on the experience of a similar absence of multiply charged species in the majority of related experiments where this type of “cutaway” source block is used.^7,72–74^

We therefore infer that Al^+^ is also the predominant charged species present in the RAS-LIF experiments, whose presence in our apparatus has been positively confirmed for the first time through the introduction of the MCP detector and deflector. The dominance of a single charged species is further consistent with the absence of sharp bimodality in the TOF distributions in Figure 3. While previous independent work has identified multiply charged Al^*n*+^ cations, at laser pulse energies comparable to the highest values used here but noting again the difficulties of comparing fluences in different experiments, the reported yield of Al^2+^ was only around 5% of that of Al^+^.^49^ The higher-charged species were progressively less abundant. Note also that the work of Torrisi et al. has shown that the multiply charged species are more strongly concentrated along the surface normal, which would mean that they are further discriminated against in the RAS-LIF geometry (although not in the source-block experiments).^49^

The Al atom TOF profiles at lower ablation fluences (see TOF distributions in Figure 6 and the derived KE distributions in the SI, Section S2, Figure S3) are qualitatively similar to those we have reported previously at comparable fluences.^14^ The faster Al component is most-strongly dominant at lower ablation fluences. We have extended the measurements here to higher fluences, finding that the KE of the faster component increases initially with laser fluence, but only modestly and not in general in proportion to the laser fluence; it changes very little between the higher fluences (see Figure 6 and Table 1). At the same time, the yield of Al neutrals increases, but also increasingly less than linearly as fluence increases, as shown in Figure 4.

A slower component to the Al KE distribution, which appears as a relatively flat, extended tail in the TOF distribution at lower fluences, increases in relative importance at higher fluences, as can be seen in Figure 6. We are not aware that such a feature has been reported previously, other than that the tail was also observed at lower fluences in our previous work,^14^ but it appears to be a genuine feature of the ablation process under our conditions. Geometric constraints prevent the products of off-axis collisions with surfaces in the apparatus at points upstream of Aperture 2 from passing through Aperture 3 and reaching the probe volume (see Figure 1b). Auxiliary LIF TOF measurements, in which the location of the probe beam was varied (see SI, Section S5, Figure S7), preclude collisions with surfaces closer to the observation point. All the available evidence is consistent with this slow peak traveling unmodified from the source region with the lower speeds implied by its TOF.

The absolute speeds of the Al atoms here are somewhat higher than we had reported previously under nominally similar conditions of 8 mJ pulse^–1^, where we found previously a peak arrival time nearer to ∼60 μs in comparison to ∼45 μs here (Figure 6).^14^ The most likely reason is again that a different ablation laser was used in our earlier work, which may have a different spatial and temporal structure from the one used here, affecting the dynamics of the ablation. We note that even the hotter components of the KE distributions here are much *colder* than those reported for Al previously by Torrisi et al, using postablation ionization and subtraction of the ionic contribution.^49^ Under the most directly comparable conditions at our highest fluences, we measure (Table 1) a mean KE of 16 eV, in contrast to ∼50 eV there. If these differences are genuine, it suggests some currently unidentified differences in the ablation conditions and/or target samples.

In contrast, we find that the Al^+^ KE distributions are generally much hotter and much more sensitive to ablation fluence (see Figure 3, Table 1, and the KE distributions in the SI, Section S2, Figure S2) than for the Al atoms. The mean Al^+^ KEs at the highest fluences are almost 2 orders of magnitude higher than those for Al. At the same time, the Al^+^ yield only increases less-than-linearly with fluence in a very similar way to that for Al (see Figure 4). Although the absolute ratio of yields of Al and Al^+^ is unknown (due to the very different methods by which they are detected), it apparently remains almost constant as a function of ablation fluence.

This inefficient coupling of the additional ablation laser energy into heating the Al neutrals, or ejecting further material, but strong effect on the energies of the Al^+^ ions, is at least qualitatively consistent with well-established descriptions of the ablation process.^1,2,10,11^ An accepted picture is that the early part of the ablation pulse is primarily responsible for local heating and ejection of the surface material. Strong thermal heating of the sample and ejection of a significant fraction of ions are characteristics of the relatively long (*i.e*. ∼5 ns) laser pulses used here.^80^ Once a plasma is formed, it absorbs the later part of the laser pulse efficiently by inverse Bremsstrahlung.^11^ This heats the plasma, primarily through direct excitation of the electrons, which couples energy into the ions, but shields the lower regions of the plasma and the underlying metal surface, curtailing further heating and ejection of material. The different spatial regions within the nonequilibrium plume can have significantly different energy distributions. Torrisi et al. have described a model in which the cations attain a substantial component of their additional KE from Coulomb repulsion once the more-mobile electrons are ejected from the nonequilibrium plasma plume.^49,50^ However, the KEs that we find for the Al^+^ ions here are much *hotter* than they report.^49^ Again under the nearest nominally comparable conditions corresponding to our highest pulse energy, their mean Al^+^ KE is ∼70 eV. In contrast, we find ∼450 eV (Table 1). The KEs at the peak of the TOF distribution are similarly very different. Assuming both results are correct, this again suggests significant differences of some kind in the ablation conditions.

Our main aim here, though, was not to investigate the mechanism of formation of the species in the ablation, but to characterize them sufficiently to be able to identify and interpret their contributions to AlF production at the IL surface. The effects of the deflector on the Al^+^ TOF distributions (Figure 5) confirm that Al^+^ can be selectively deflected away from the MCP detector, with higher fields being required to deflect the faster ions, as expected. More importantly from the current perspective, they confirm that the ionic species can be prevented from reaching the IL surface (situated at a greater distance from the deflector and beyond a small aperture – recall Figure 1b). This has allowed us to successfully distinguish the contributions from neutral Al and ionic Al^+^ to neutral AlF production, as detected by LIF.

One of the major new observations that we present here is that Al^+^ is responsible for a substantial fraction of the AlF produced from [C_2_mim]­[Tf_2_N], as demonstrated in Figures 7 and 8. The production of neutral AlF from Al^+^ obviously involves a net overall charge transfer, but the point at which this happens, and the form of the reaction mechanism, remains undetermined.

As noted, we do not have a reliable way of estimating absolute ratios of incident Al^+^ and Al, so cannot determine their relative efficiencies for producing AlF. We can, however, say that the ratio of AlF from the two projectiles (considering only the faster component of the Al atoms) does not change significantly between the two highest fluences, as shown in Figure 4. Around 66% of the observed AlF is produced from Al^+^ at both fluences. The yield of both Al and Al^+^ increases by a factor of ∼1.5 between 8 and 30 mJ pulse^–1^, while over the same range the yields of AlF from both Al and Al^+^ increase by a factor of ∼ 3. This approximate doubling of the AlF yield per incident projectile is not too surprising considering the relatively modest changes to the KE distribution for Al atoms, but it is remarkably small given the very large increase in KE of the Al^+^ ions. This perhaps simply reflects that the overwhelming majority of the KE distributions of the Al^+^ ions lie substantially above the C–F bond energy of ∼ 5.5 eV in the fluoroalkyl −CF_3_ group in [Tf_2_N]^−^.^81–83^ It does also suggest that the branching to the very many other channels that are thermodynamically accessible for Al^+^ ions with KEs of several 100 eV is not highly sensitive to the energy in this range.

The presence of the slow peak in the incident Al TOF distribution (see Figure 6, or the density-flux-transformed version in the SI, Section S2, Figure S3) does allow some approximate limits to be estimated on the threshold energy for AlF production from Al atoms. There is no sign of a corresponding later peak in AlF production within the signal-to-noise in Figures 7 or 8, which indicates that these Al atoms are minimally reactive to produce AlF compared to those in the faster peak. As noted above, the KE corresponding to the peak of the slower wave at ∼180 μs is ∼100 kJ mol^–1^ (1.0 eV) while, for comparison, the minimum between the peaks at ∼100 μs corresponds to ∼320 kJ mol^–1^ (3.2 eV). This bracket of ∼1–3 eV seems reasonable in the context of the relatively little that is known about these types of reaction. There are no available data for Al reactions with fluoroalkyl species in the gas phase with which to compare, which in itself reflects the likely threshold energy being a significant fraction of the C–F bond energy and hence inaccessible under thermal or even modestly superthermal conditions. We had previously speculated, by extrapolation from Al reactions with more-weakly bonded NF_3_ and SF_6_, that the threshold energy for C–F bonds was likely to be at least 60 kJ mol^–1^ (0.6 eV).^14^

The second major new insight gained here is on the KE distribution of the AlF leaving the IL surface. This was ambiguous in our previous work, because we were unaware at that time that the majority source of the AlF was the (unobserved) Al^+^ rather than the (observed) Al atoms.^14^ Consequently, previous arguments based on the appearance time of the AlF at a single probe position were not a reliable guide to AlF speeds because they were not based on the correct, earlier arrival time of the Al^+^ at the surface. We have resolved that here through the separation of the AlF into its contributions from Al^+^ and Al, as shown in Figure 8 alongside the distributions of arrival times for the two projectiles. As noted above, there are a number of difficulties associated with attempting to reproduce the AlF profiles by convoluting these arrival distributions with assumed speed distributions for the AlF leaving the surface, particularly given the short flight path relative to the dimensions of the dosed area and length of the LIF probe volume. These are avoided, though, by probing the AlF at a sequence of distances, as shown in Figure 9. This demonstrates unequivocally that the majority of the AlF has speeds compatible with a thermal distribution at the temperature of the IL surface (320 K), for both Al^+^ and Al projectiles.

Finally, we reflect on the implications of these new results for the potential use of this form of RAS-LIF as a surface-sensitive probe. The high KE of Al^+^, especially, is *a priori* consistent with significant penetration of the Al^+^ into the liquid, accentuated by the fact that we now know the Al^+^ KEs are significantly higher under our conditions than would have been anticipated from previous related work.^49^ This assertion of significant penetration is based on the well-known observation of inelastic scattering of ions from similar ILs and other organic liquids at only moderately higher KEs in related techniques such as LEIS and NICISS.^58–64^ The inelastic energy loss as a function of distance into the material is, for example, the basis of depth-profiling using NICISS, for which probe depths up to several tens of Å are reported.^64,84^ The most direct evidence from the work here itself is that the AlF KE distributions do not carry any clear signature of the high incident KE, particularly for Al^+^, which produces the majority of the AlF, at least for [C_2_mim]­[Tf_2_N] under our conditions. This energy is presumably largely dissipated through secondary interactions of the AlF with its environment before escaping into the gas phase. Although it is still a subject of active debate whether molecules desorbing from liquid surfaces will necessarily have internal distributions that exactly match the temperature of the liquid,^85^ nevertheless the near-thermal rotational distributions observed here (Figure 10) and previously also corroborate the conclusion that the majority AlF has experienced multiple interactions before leaving the liquid surface.^14^

The only contrary residual evidence of the AlF having been formed in a high-energy process is the minor component of vibrationally excited AlF. As shown for the first time here in Figure 10, this is associated only with the rising edge of the AlF appearance profile. Note that although the rising edge is entirely produced by reaction of Al^+^, so is the great majority of the AlF at the peak which does not show the same degree of vibrational excitation. This indicates that the correlation is between vibrational excitation and higher speeds of the AlF and not simply production from Al^+^. Vibration is in general very well-known to be more robust to relaxation in secondary collisions than either rotation or translation, so this is some indication that a proportion of the AlF is formed in the outer regions of the IL and escapes before being fully thermalized.

What remains unknown is the efficiency with which AlF may be lost through secondary reactive processes in the IL environment. As noted in the Introduction, if this were rapid then it would mean that this RAS-LIF method would still have a high degree of surface sensitivity. The observation of near-thermalized AlF suggests that it does not react with high efficiency, at least in the case of [C_2_mim]­[Tf_2_N]. The reactivity of AlF more generally in fluorinated media is largely unknown, although it has been suggested that in the laser detonation of Al nanoparticles embedded in polytetrafluoroethylene AlF is produced and then lost in secondary reactions to form polyfluoroaluminum species.^86^ However, such secondary reactions are likely to be dependent on the specific chemical composition of the medium. This may be one among a number of possible explanations for the anomalously low AlF yields that were observed, particularly for ILs containing [OTf]^−^, in our previous work.^15^

Nevertheless, the broader interesting new insight gained here is that there are two independent probe projectiles (Al and Al^+^) both of which produce AlF when they interact with an IL surface. As yet, their relative contributions have only been determined for one IL in which F is only present at a −CF_3_ site in the [Tf_2_N]^−^ anion. It would be potentially interesting to explore the differential reactivity of these two projectiles in a wider range of ILs containing fluorine in different chemical environments.

## Conclusions

Laser ablation of an Al metal target produces both Al and Al^+^, as previously established, but with substantially higher Al^+^ kinetic energies than was anticipated. Multiply charged Al^*n*+^, or other cluster or oxide cations, are not a significant fraction of the cation yield under our conditions.

Both Al and Al^+^ react to produce AlF at the surface of the prototypical ionic liquid, [C_2_mim]­[Tf_2_N]. Under our conditions, Al^+^ is responsible for the majority (∼66%) of the yield of AlF. The AlF leaves the IL surface with near-thermal kinetic energy and rotational distributions. This is consistent with significant penetration of the projectiles into the liquid and thermalization of the AlF in secondary interactions before escaping. The only residual indication of the high-energy process through which it is formed is a component of vibrationally excited AlF, which is correlated with the fastest products.

The efficiency of secondary reactions of AlF, which may still convey surface sensitivity to this form of the RAS-LIF method, and its dependence on the chemical composition of the liquid, remains to be established.

## Supplementary Material



## Notes

Source-block ablation experimental apparatus; TOF, speed, and kinetic-energy distributions for Al^+^ and Al; integrated fluxes of incoming Al species and outgoing AlF number densities; predicted transmission of ions as a function of applied electric field on the deflector; Al atom LIF appearance profiles at different probe-laser positions; empirical fitting of peak positions in AlF appearance profiles; prediction of rising-edge positions in AlF appearance profiles (PDF)

## ∥

Western Australian School of Mines, Curtin University, Bentley WA 6102, Australia
