# Supplementary material for: Reaction of a Laser-Ablated Al Plume with a Fluorinated Ionic-Liquid Surface: Characterizing the AlF-Producing Species
Source: J Phys Chem C Nanomater Interfaces. 2026 Aug 7;130(33):11618–33. doi: 10.1021/acs.jpcc.6c03846 (PMC13501957; doi:10.1021/acs.jpcc.6c03846)
Supplement: Supplementary file 1 [file jp6c03846_si_001.pdf]

# Reaction of a Laser-Ablated Al Plume with a Fluorinated Ionic-Liquid Surface: Characterizing the AlF-Producing Species

*Philip A. J. Pearcy,* [(a)] *Paul. D. Lane,* [(a)] *Peter T. Rubli,* [(b)] *Christian T. Haakansson,* [(b)] *Peter D. Watson,* [(b)]† *Stuart R. Mackenzie,* [(b)] *Naomi S. Elstone,* [(c)] *Duncan. W. Bruce,* [(c)] *John M. Slattery,* [(c)] *Matthew L. Costen,* [(a)] *Kenneth G. McKendrick* [(a)]*.

(a) Institute of Chemical Sciences, School of Engineering and Physical Sciences, Heriot-Watt University, Edinburgh, EH14 4AS, UK

(b) University of Oxford, Department of Chemistry, Chemistry Research Laboratory, Mansfield Rd, Oxford OX1 3TA, UK

(c) Department of Chemistry, University of York, Heslington, York YO10 5DD, UK

†Present address: Western Australian School of Mines, Curtin University, Bentley, WA 6102, Australia

## Supporting Information

**Table of Contents**

S1. Source-block ablation experimental apparatus.

S2. TOF, speed and kinetic-energy distributions for $Al^+$ and Al.

S3. Integrated fluxes of incoming Al species and outgoing AlF number densities.

S4. Predicted transmission of ions as a function of applied electric field on the deflector.

S5. Al atom LIF appearance profiles at different probe-laser positions.

S6. Empirical fitting of peak positions in AlF appearance profiles.

S7. Prediction of rising-edge positions in AlF appearance profiles.

S8. References for Supporting Information

## S1. Source-block ablation experimental apparatus

Laser ablation of a metal target within an expanding carrier gas was carried out following well-established principles.[1-4]

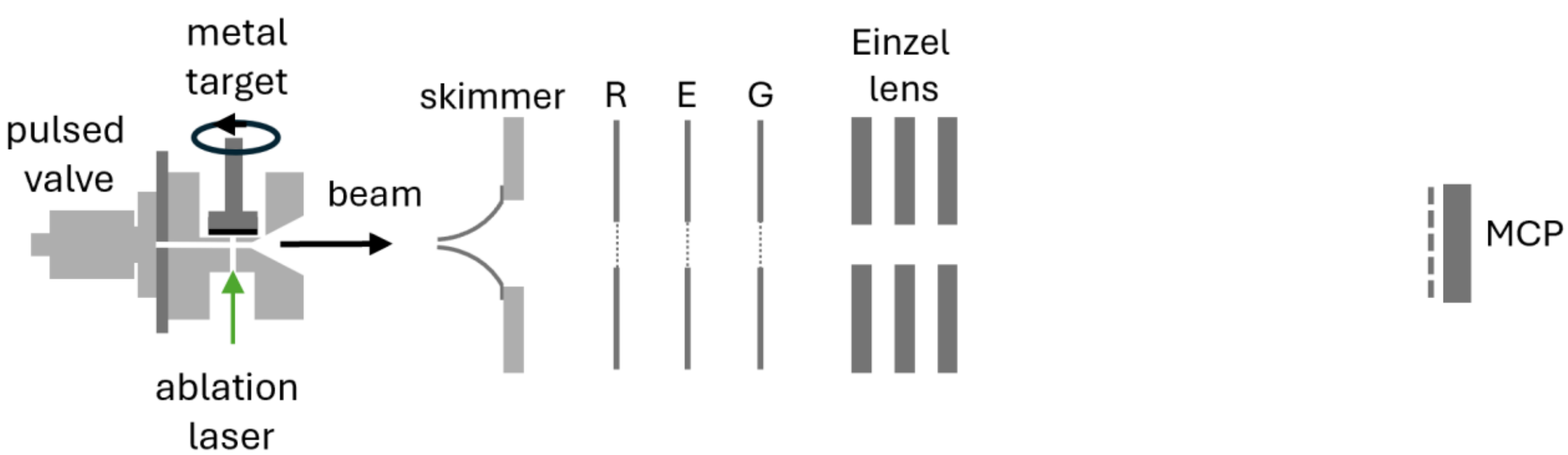


Figure S1: Schematic of the source-block apparatus used to record the TOF mass spectra of He-entrained $Al^+$. The $Al^+$ ions are produced in the cutaway source by ablating the rotating metal disc target using a 532 nm laser pulse within a He gas pulse. The $Al^+$ is then accelerated within the Wiley-McLaren (WM) ion optics arrangement (made up of repeller (R), extractor (E), and ground (G) plates) towards the retractable MCP detector. An additional Einzel lens focuses the $Al^+$ beam onto the MCP detector.

As shown in Figure S1, a rotating Al metal-disc target was ablated within a cutaway source[1] using a focused (lens focal length = 300 mm) 532 nm pulse from a Nd:YAG laser (Quantel Ultra, 8 ns pulse, 3-4 mJ per pulse). A Li:Al (1:9 atomic ratio alloy) target was also used to confirm the mass calibration and the sensitivity of the method to species with lower *m*/*z*. Ablation took place in the throat of a pulsed supersonic expansion of the He carrier gas (delivered via a solenoid valve from

a backing pressure of 2-5 bar). The resulting molecular beam was skimmed before ions were accelerated using a Wiley-McLaren (WM) extraction setup,[5] orientated to accelerate ions through the chamber along its central axis. The ions travelled through an Einzel lens, before being detected using an MCP detector assembly 600 mm away from the midpoint of the repeller and extractor plates of the WM optics. The signal from the MCP anode was recorded on an oscilloscope, with time zero being defined as the trigger for the pulsed WM plates. The TOF spectrum was converted into a mass spectrum using values acquired from a calibration curve to deduce the mass of the constituent parts of the molecular beam.

## S2. TOF, speed and kinetic-energy distributions for $Al^+$ and Al.

The measured TOF distributions of $Al^+$ ion flux (compare Figure 3 of the main text), $F_t(t)$, are reproduced in Figure S2 a). They were transformed to speed distributions, $F_v(v)$, through the relationship

$$F_v(v)\mathrm{d}v = F_t(t(v))\left|\frac{t^2}{z}\right| \mathrm{d}v \qquad \text{Eqn (S1)}$$

including the usual Jacobian factor, where $t$ is the measured TOF at distance, $z$, between ablation point and MCP detector and $v$ is the speed. The results are shown in Figure S2 b). They were further transformed to kinetic-energy distributions, $F_E(E)$, through

$$F_E(E)\mathrm{d}E = F_v(v(E))\frac{1}{mv}\mathrm{d}E = F_t(t(E))\left|\frac{t^3}{mz^2}\right|\mathrm{d}E \qquad \text{Eqn (S2)}$$

where $m$ is the mass of $Al^+$, with the results in Figure S2 c).

The measured TOF distributions, $n_t(t)$, of Al atom number density (compare Figure 6 of the main text) were first converted to flux distributions, $F_t(t)$, by weighting with the speed

$$F_t(t)\mathrm{d}t \propto v\, n_t(t)\,\mathrm{d}t = \frac{z}{t} n_t(t)\,\mathrm{d}t \qquad \text{Eqn (S3)}$$

where $t$ is the measured TOF at distance, $z$, between the ablation point and the LIF detection zone. The results are shown in Figure S3 a). They were transformed to speed (Figure S3 b)) and KE (Figure S3 c)) in the same way as the $Al^+$ distributions via equations (S1) and (S2), respectively.

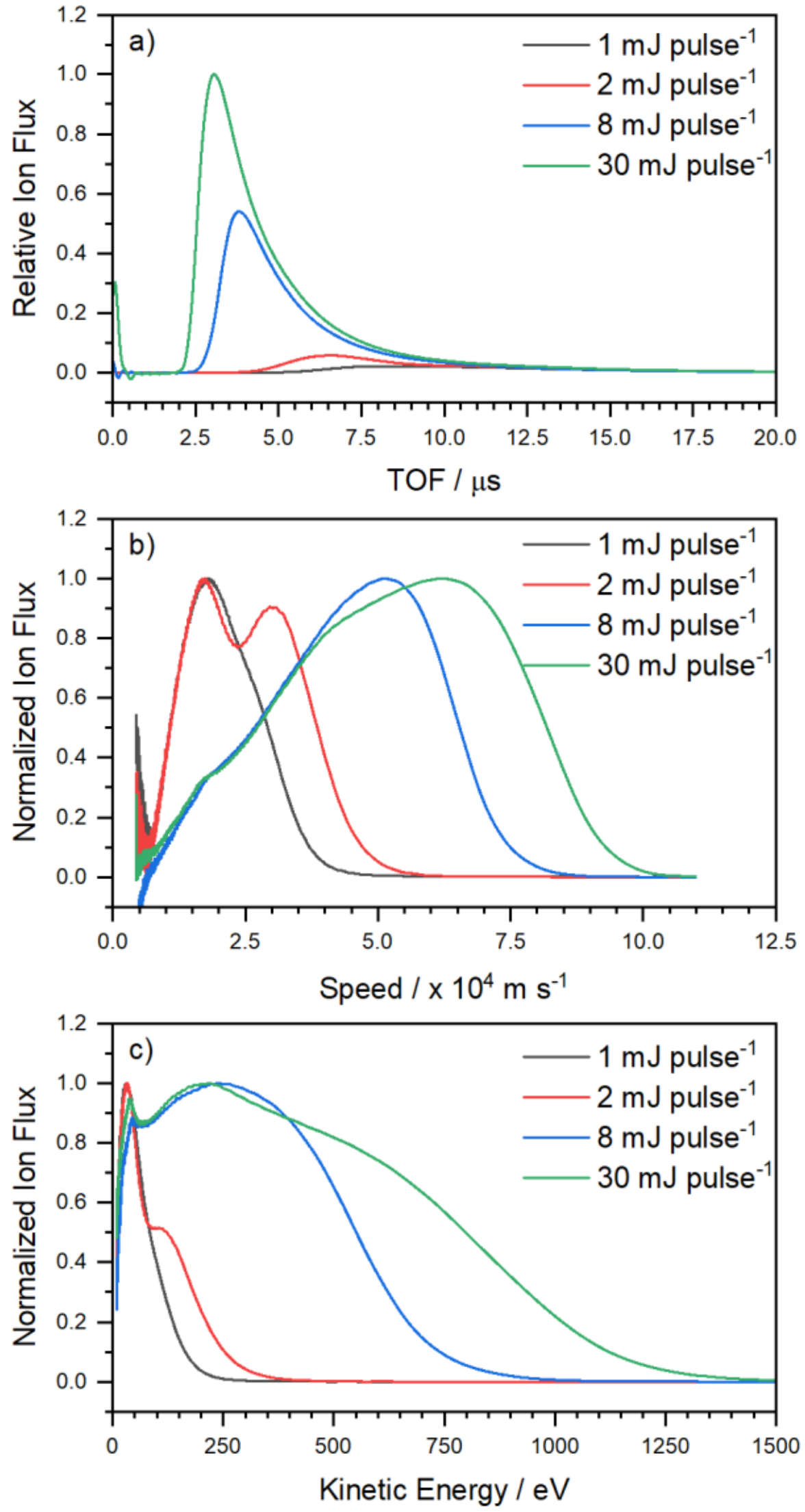


Figure S2: a) TOF profiles for $Al^+$ for a range of ablation laser pulse energies (1, 2, 8, and 30 mJ), as recorded on an MCP detector situated 220 mm away from the point of ablation. They were transformed to b) speed and c) kinetic-energy distributions via equations (S1) and (S2). The profiles in a) are scaled relative to the intensity of the 30 mJ $pulse^{-1}$ data; the speed and KE distributions are internally normalized relative to their maximum values.

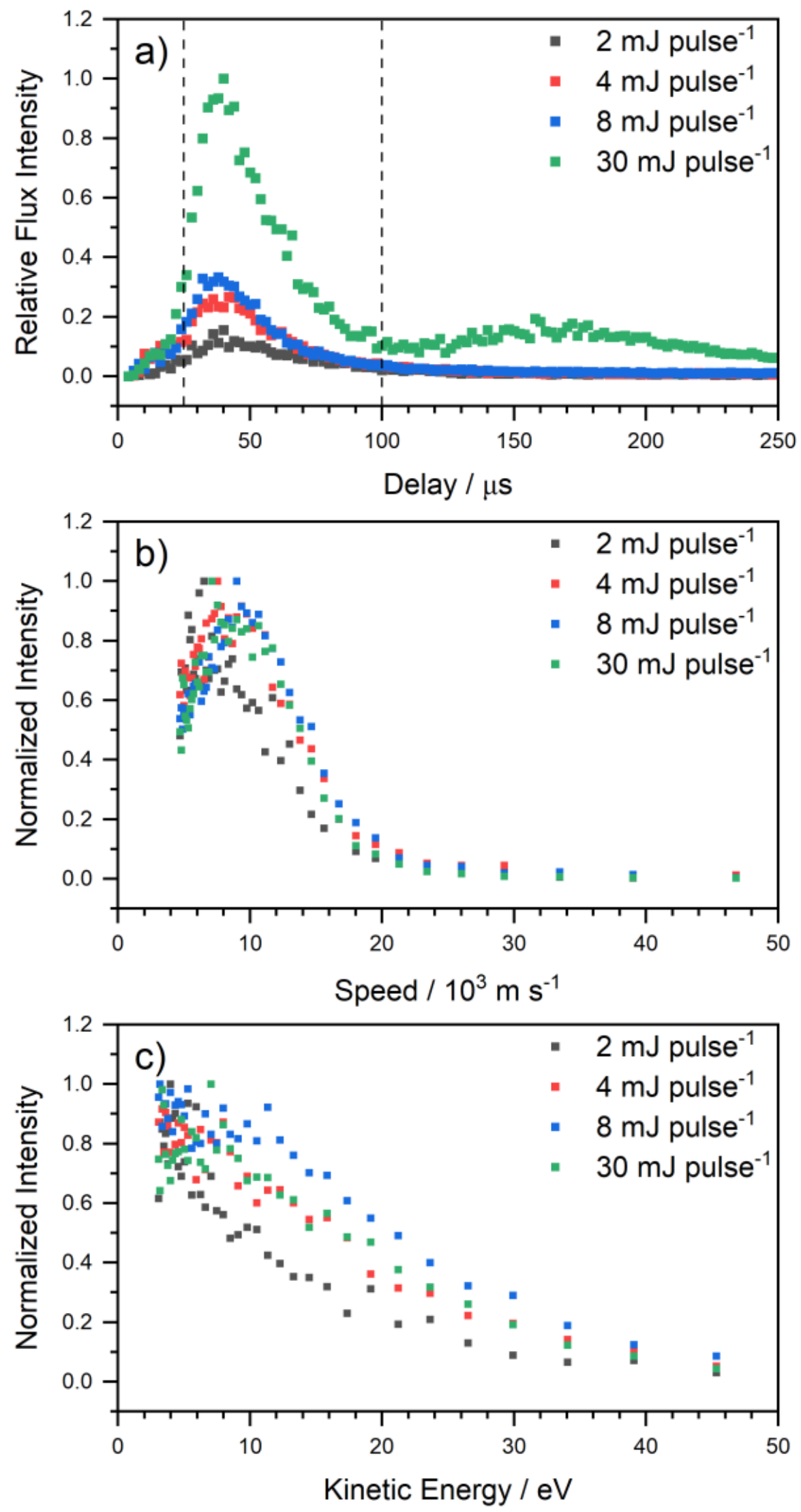


Figure S3: a) Al flux TOF profiles at different ablation-laser pulse energies (2, 4, 8, and 30 mJ). They were derived from LIF number-density profiles via equation (S3). The b) speed and c) kinetic-energy distributions result from the Jacobian transformations in equations (S1) and (S2). Results in b) and c) are only shown between limits of 20 µs and 100 µs (black dashed lines in a)); they became numerically unstable at the shortest delays and, for the reasons explained in the main text, the secondary maximum at longer delays was excluded. Profiles in a) are scaled relative to the intensity of the 30 mJ $pulse^{-1}$ data; the speed and KE distributions are internally normalized relative to their maximum values.

## S3. Integrated fluxes of incoming Al species and outgoing AlF number densities

The numerical values of the integrated quantities represented in Figure 4 of the main text are listed in Table S1.

Table S1: Integrated fluxes of incoming Al species ($Al^+$ and Al) and integrated AlF number densities produced by reaction of each of them at the IL surface, as a function of ablation-laser energy. Uncertainties are 1σ standard errors in the mean of repeated measurements.

| | **Incoming Al species**[a] | | **Outgoing AlF**[b] | |
|---|---|---|---|---|
| **Pulse energy / mJ pulse$^{-1}$** | **$Al^+$** [c] | **Al** [d] | **AlF from $Al^+$** [e] | **AlF from Al** [e] |
| 1 | 0.124 ± 0.001 | - | - | - |
| 2 | 0.234 ± 0.001 | 0.285 ± 0.082 | - | - |
| 4 | - | 0.549 ± 0.127 | - | - |
| 8 | 1.000 ± 0.008[f] | 1.000 ± 0.294[f] | 1.000 ± 0.069[f] | 0.500 ± 0.087[g] |
| 30 | 1.644 ± 0.006 | 1.744 ± 0.412 | 3.303 ± 0.188[h] | 1.684 ± 0.138 |

(a) Relative integrated fluxes of incoming species.

(b) Relative integrated AlF number densities. Note the comment in the main text that the speed distributions of the AlF produced by $Al^+$ and Al are very similar, and so the ratio of fluxes will be very similar to the ratio of number densities.

(c) See Figure 2 of the main text.

(d) See Figure S3 a). The integration is over the faster component (20 - 100 μs) only.

(e) See Figures 7 and 8 of the main text.

(f) Results for $Al^+$, Al and AlF from $Al^+$ are independently normalized to their values at an ablation pulse energy of 8 mJ.

(g) As measured relative to AlF from $Al^+$.

(h) The ratio of AlF signals at 8 and 30 mJ pulse has been set to the accurate value determined previously.[6]

## S4. Predicted transmission of ions as a function of applied electric field on the deflector.

The parameters relating to the electrostatic deflector are defined in the schematic diagram in Figure S4.

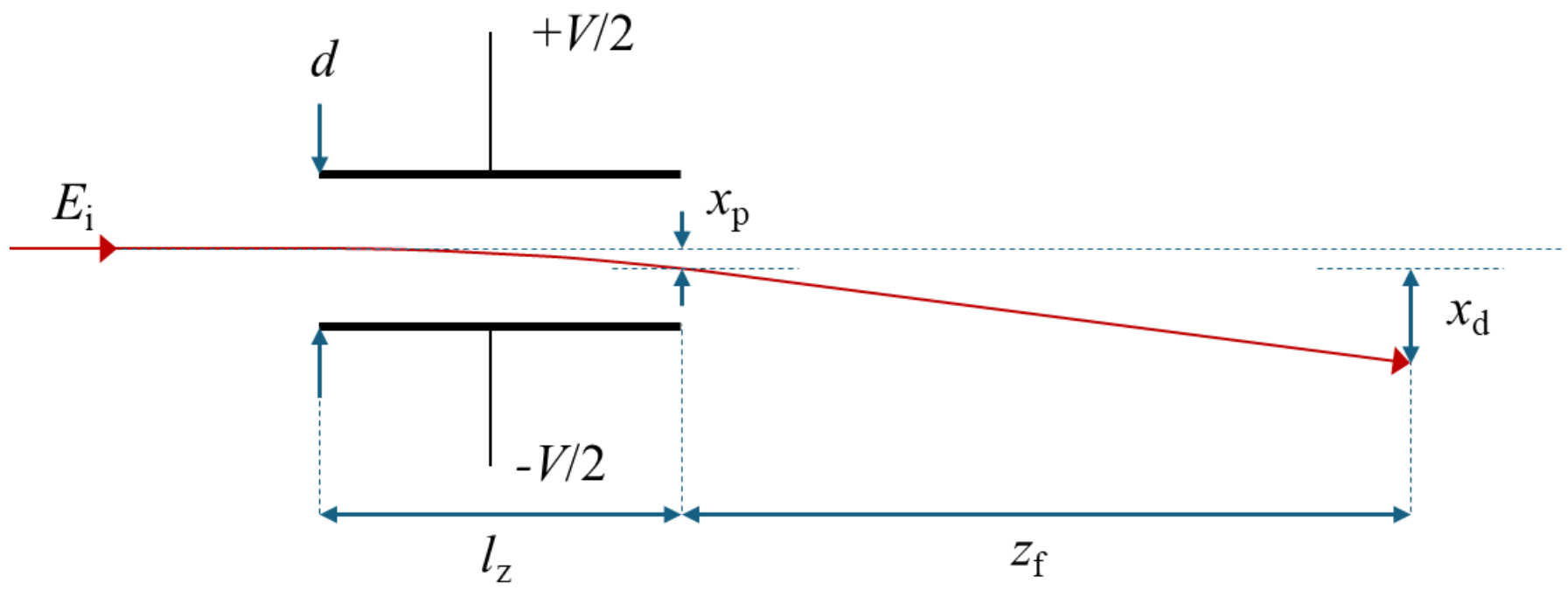


Figure S4: Schematic diagram of the operation of the deflector. Equal but opposite voltages, $\pm V/2$, were applied to the parallel plates, creating a variable electric field of magnitude, $\underline{E} = V / d$. A schematic cation trajectory is indicated in red. The other quantities are as defined in the text.

The initial KE along the $z$ direction is $E_\mathrm{i} = \frac{1}{2} m v_\mathrm{i}^2$, where $m$ is the mass of the ion and $v_\mathrm{i}$ is the initial speed. The ion passes between a pair of parallel plates of length, $l_z$ = 20 mm, and separation, $d$ = 10 mm. It travels a further distance, $z_\mathrm{f}$, after leaving the deflector before reaching a limiting aperture. The dimensions of the aperture are determined either by the active area of the MCP detector (18 mm diameter) for ion TOF measurements or by Aperture 3 (10 mm diameter; with $z_\mathrm{f}$ = 240 mm) for reactive scattering at the liquid surface (see Figure 1 b) of the main text).

The displacement along the $x$ direction, $x_\mathrm{d}$, due to linear off-axis flight after leaving the deflector (neglecting the effects of fringing fields) for a singly charged cation, such as $Al^+$, with elementary charge, $q$, is

$$x_{\mathrm{d}} = \frac{q\,V\,l_{\mathrm{z}}\,z_{\mathrm{f}}}{m\,v_{\mathrm{i}}^{2}\,d} \qquad \text{Eqn (S4)}$$

There is also, in principle, a deflection due to curvature of the ion trajectory within the plates, $x_{\mathrm{p}}$, given by

$$x_{\mathrm{p}} = \frac{q\,V\,l_{\mathrm{z}}^{2}}{2\,m\,v_{\mathrm{i}}^{2}\,d} \qquad \text{Eqn (S5)}$$

In practice, $x_{\mathrm{p}}$ is the minor contribution when $z_{\mathrm{f}} > 2l_{\mathrm{z}}$, which is true here for MCP detection (where $z_{\mathrm{f}} = 60$ mm) and, especially for reactive scattering at the liquid ($z_{\mathrm{f}} = 240$ mm), and it is not in any case significant for the version of the estimates that are made below.

The $Al^{+}$ KEs that we determine from the ion TOF (see Figure S2 and Table 1 in the main text) extend out to $E_{\mathrm{i}} = 1500$ eV at the highest ablation pulse energy of 30 mJ. The deflector potential differences, $V$, that were applied to deflect the ions away from the MCP detector spanned 0 to 250 V (see Figure 5 of the main text). There will not be a sharp cut off in the ion energies, and hence TOFs, that are transmitted at each deflection voltage because of the spatial spread transverse to the direction of travel, and potential divergence, of ion trajectories that pass through Apertures 1 and 2. Nevertheless, it is possible to make a reasonable approximate estimate of the maximum deflection that would just allow an ion to hit the outer edge of the detector as the 14 mm sum of the radius of Aperture 2 (5 mm) and the radius of the active area of the MCP detector (9 mm). This assumes that an ion just misses the inner (*i.e.* negative $x$) edge of Aperture 2 and the outer (positive $x$) of the detector. If this is equated to $x_{\mathrm{d}}$ (*i.e.* the deflection due to off-axis velocity between Aperture 2 and the MCP detector) then the *minimum* KEs that would just hit the detector can be calculated from Eqn (S4). These have been converted to the corresponding *longest* TOF values that should hit the detector. They are displayed in Figure S5, which is a reproduction of Figure 5

of the main text with these estimated limits added as dashed lines, using the same color code for deflection voltages.

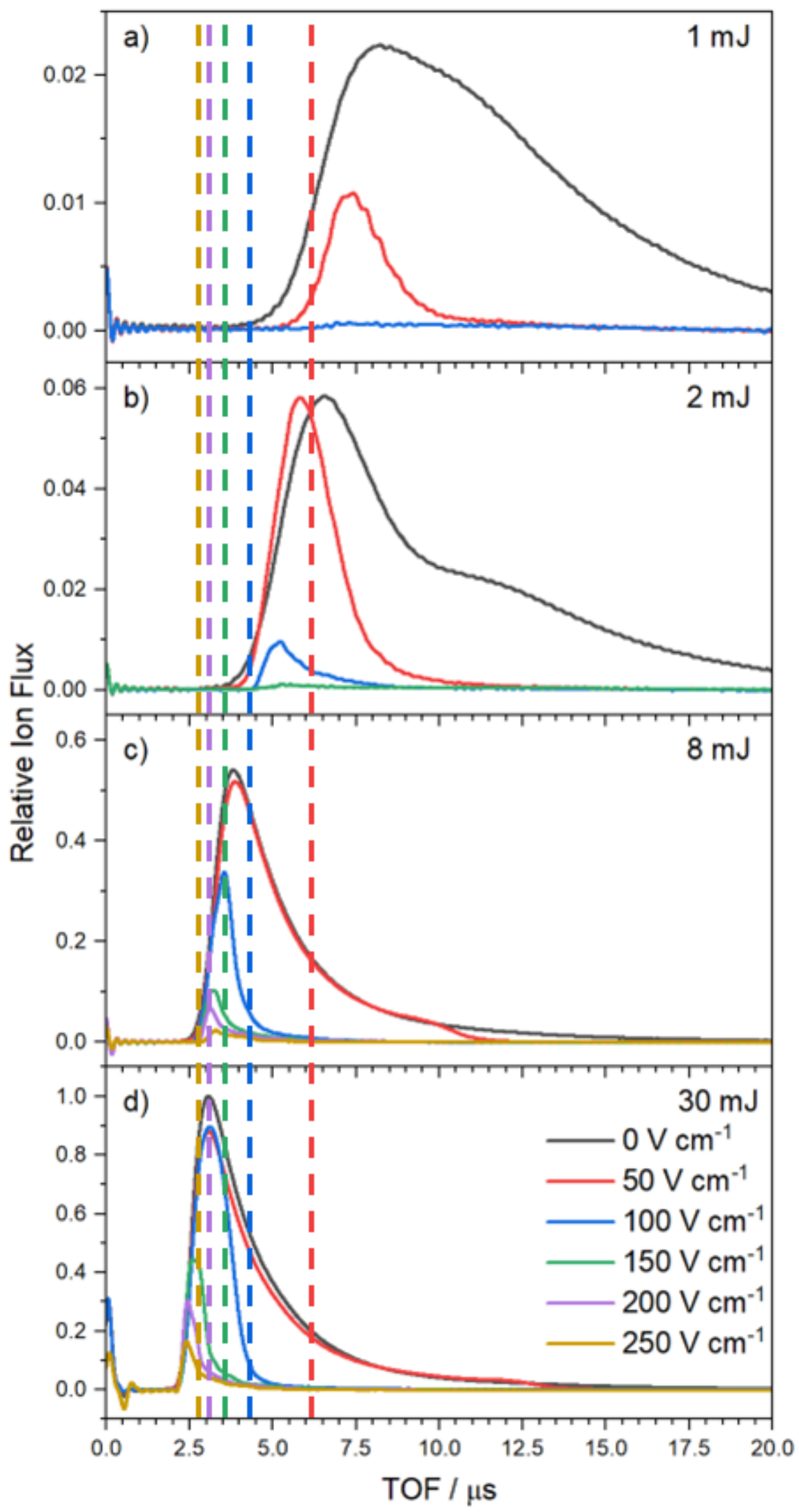


Figure S5: Effect of the deflector on the TOF profiles for $Al^{+}$ across a range of ablation laser pulse energies, as indicated (compare Figure 5 of the main text). The vertical dashed lines are approximate estimates of the longest TOF values that would just hit the detector at different deflector field strengths, using the same color code as in the legend in d).

As can be seen in Figure S5, the approximate limits are broadly plausible, especially for the faster ions at shorter TOFs which require higher ablation energies to be produced in measurable amounts. There is, however, some ‘leakage’ of ions with longer TOFs than is predicted, particularly at lower deflector voltages and longer times of flight. There is also some dependence on the ablation pulse energy of the TOFs which are effectively deflected by a given voltage difference.

There are a number of potential explanations for these discrepancies. If the charge density in the ion packet is sufficiently high, space-charge effects could cause significant continued Coulomb repulsion beyond the deflector region. The spatial expansion lateral to the direction of travel would then cause some trajectories to hit the detector that would otherwise have missed based on straight-line predictions. This would be consistent with the discrepancies being largest for the slowest ions, which are most susceptible to space-charge effects. It would also be compatible with the observed higher proportion of the ions surviving beyond the predicted 50 V $cm^{-1}$ cut-off at higher ablation pulse energies, also correlated with higher ion densities. If present, this effect would likely also cause some spatial expansion along the direction of travel and hence some broadening of the TOF distribution, which would be difficult to distinguish from a spread of speeds in the source region, although any effects on the average KE would largely cancel.

A related but distinct effect could arise if the time spent in the source region prior to being accelerated due to Coulomb expansion is a significant fraction of the observed TOF at the MCP detector. This would mean that the KEs of ions as they pass through the detector are higher than they appear on the basis of the total TOF and so they would be less-efficiently deflected than predicted. In that case, their observed TOF leads to underestimate of the true KE as experienced at the IL surface.

A further alternative to space-charge effects is that the plasma created by the ablation penetrates into the deflection region. In that case, there may be some partial shielding of the ions from the electric field and hence reduced deflection. This would also be consistent with larger discrepancies at higher ablation-pulse energies, which we would expect to lead to more spatially extended plasmas.

We have not attempted to investigate further which, if any, of these effects may be responsible for the observed effects of the deflector on signals at the MCP detector. However, we reiterate that the primary aim of these studies was to establish whether ions could be successfully prevented from reaching the IL surface in RAS experiments. If they can be successfully deflected off the MCP detector, the relative geometries make this highly likely: the ratio of distances, $z_f$, from the end of the deflector to Aperture 3 and to the MCP detector, respectively, is 240 mm / 60 mm = 4. The off-axis deflections, $x_d$, at the two locations will be in the same proportion. Furthermore, Aperture 3 is also only 10 mm in diameter compared to 18 mm for the MCP detector. We are therefore very confident that even the fastest ions, which only just reach the MCP detector at the higher deflector voltages, will be prevented from reaching the IL surface under the same conditions. This was confirmed by the absence of significant further change in AlF production beyond a field strength of around 80 V cm$^{-1}$, as shown in Figure 7 of the main text. To reinforce this, additional separate measurements were made to higher field strengths, as shown in Figure S6. The absence of significant further change between 100 and 200 V cm$^{-1}$, with no detectable AlF signal at delays below ~40 µs exclusively associated with $Al^+$, confirmed that all the $Al^+$ had been effectively removed by that point.

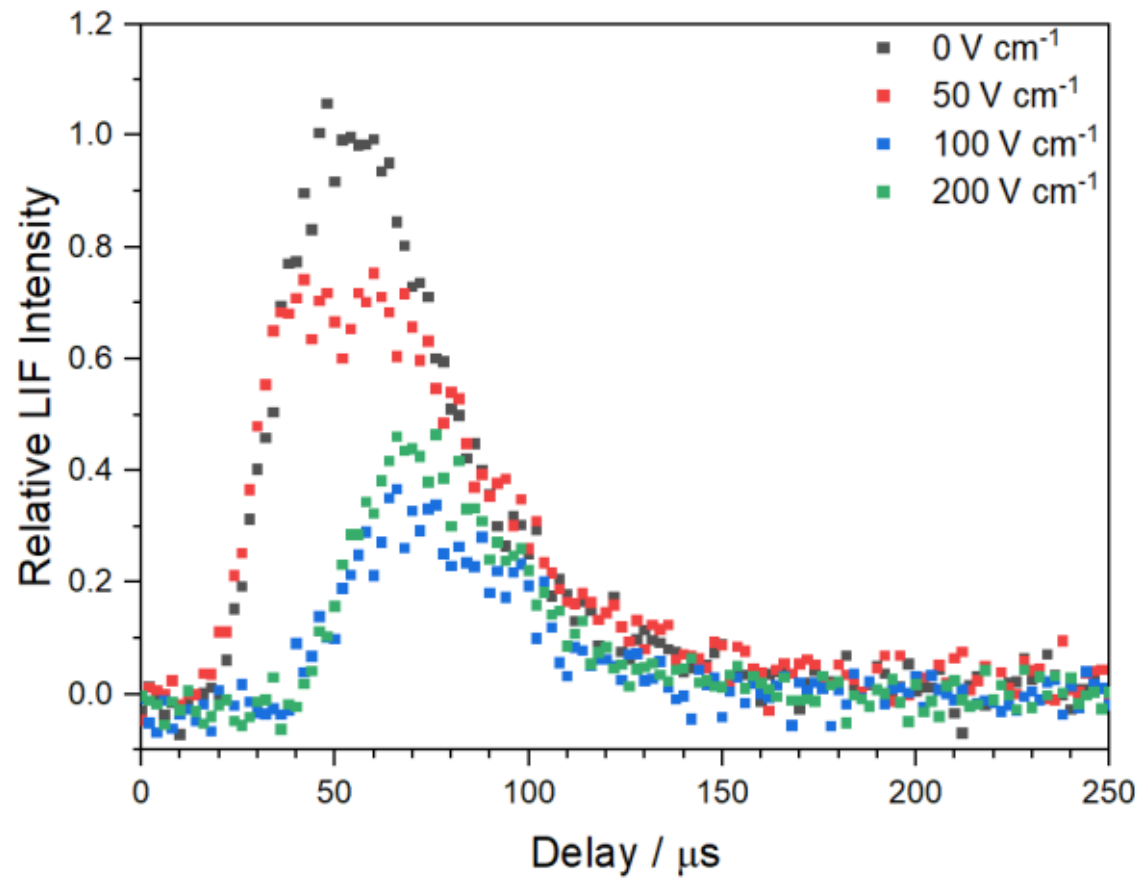


Figure S6: LIF appearance profiles of AlF produced *via* reactive scattering of Al species on the [$C_2$mim][$Tf_2$N] liquid surface. The profiles were recorded with an ablation pulse energy of 30 mJ, and across a range of deflector electric field strengths (0-200 V $cm^{-1}$). The data are normalized relative to the peak at 0 V $cm^{-1}$ (black squares).

## S5. Al atom LIF appearance profiles at different probe-laser positions.

Al atom LIF appearance profiles were also recorded at different probe-laser positions along the incident direction of travel, as shown in Figure S7. They are labelled by the distance that the probe position would be from the IL-coated wheel, because in practice this was the defined reference point in the laboratory, but as in Figure 6 of the main text, no wheel was present for these measurements. The set of probe-laser positions is the same as those used for detection of the returning AlF in Figure 9 of the main text.

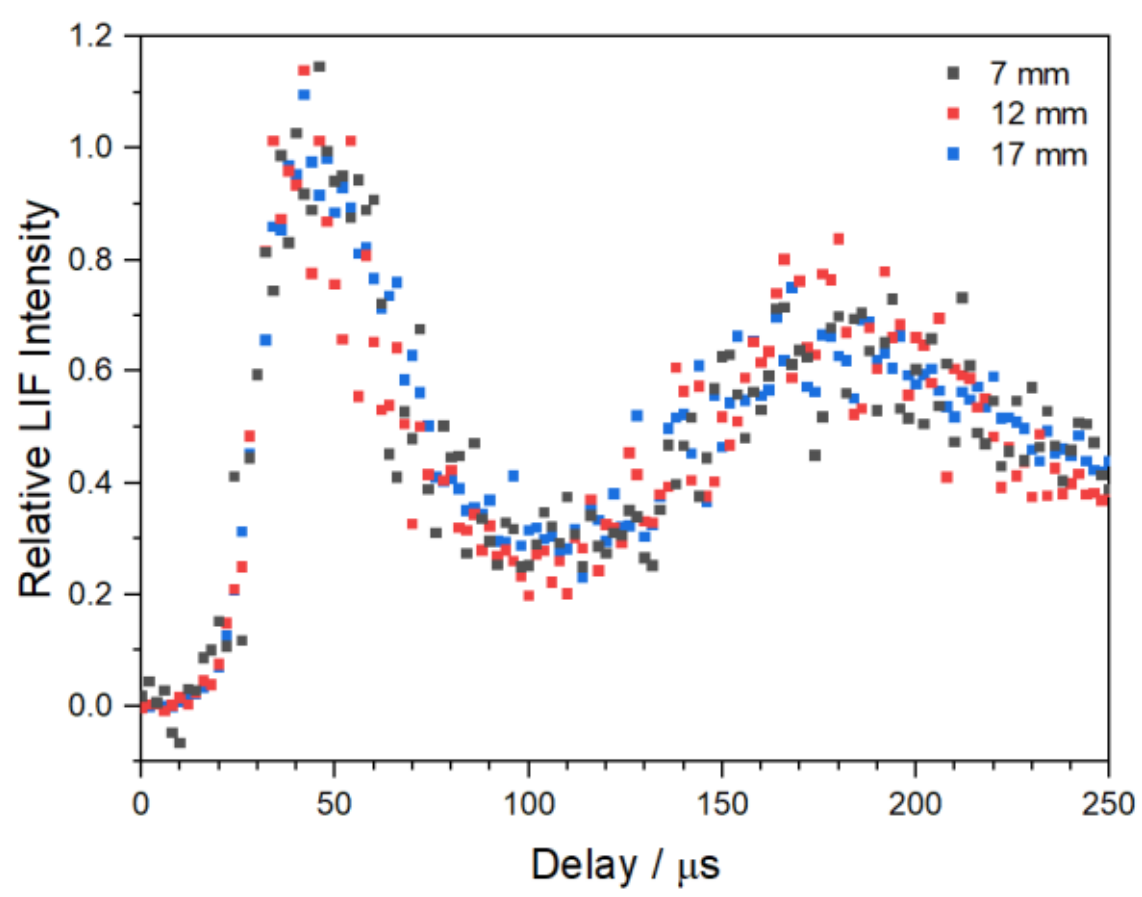


Figure S7: Al atom LIF appearance profiles recorded at different surface-to-probe distances, as indicated (the color scheme is the same as that in Figure 9 a) of the main text). The profiles were recorded using an ablation pulse energy of 30 mJ, with no applied deflector field.

As can be seen from Figure S7, the Al LIF appearance profiles changed very little over the 10 mm variation in probe position. This is consistent with these changes being a small fraction of the total distance travelled (= 470 mm at the central probe-surface distance of 10 mm) from the laser-ablation source. The expected very small proportional shifts are within the scatter due to the signal-to-noise of the measurements; note that they would be towards longer TOFs for shorter distances

(as defined). This confirms that neither of the main features, and in particular the secondary peak at longer times, can be the result of inelastic scattering of Al atoms, or $Al^{+}$ neutralization, at a surface in the apparatus close to the observation point. If this were to be the case, the additional delay of over 100 μs between the earlier and later peaks in Figure S7 would be the result of the substantially curtailed speed of the Al after it left the relevant nearby surface. The 10 mm variation in the location of the LIF probe beam would be a significant fraction of the distance travelled following the collision and hence the position of the secondary peak would have been observed to shift significantly. The absence of any such variation in Figure S7 can be contrasted with the behavior in Figure 9 of the main text, where significant shifts (to shorter TOFs for shorter surface-to-laser-probe distances) are readily apparent for AlF produced at the IL surface.

## S6. Empirical fitting of peak positions in AlF appearance profiles.

The phenomenological fitting function used to locate the peak positions of the AlF LIF appearance profiles in Figure 9 a) of the main text, as well as their associated errors, was a polynomial-modulated Gaussian:

$$f(t) = At^2 \exp\left(-\frac{(t-t_0)^2}{2\sigma^2}\right), \qquad \text{Eqn (S6)}$$

which was found to be a sufficiently flexible functional form to allow unbiased fits in the vicinity of the observed peaks in the profiles. Physical meanings are not necessarily ascribed to the parameters but, mathematically, $A$ is an overall scaling factor (which can be adjusted so that the peak maximum is normalized to 1), $t$ is the time (as defined by the input LIF appearance profile), $t_0$ allows the central position of the Gaussian envelope to be adjusted (although note that it is not equivalent to the peak position due to the $t^2$ weighting of the leading term), and $\sigma$ is a Gaussian-width parameter.

The best-fit values for $A$, $t_0$, and $\sigma$ were derived through a standard nonlinear least-squares optimization. The desired peak positions, $t_{\text{peak}}$, used in the construction of Figure 9 b) in the main text, correspond to the best estimates of the times at which the fitted function reaches its maximum value, with its associated uncertainty ($1\sigma$ standard deviation).

## S7. Prediction of rising-edge positions in AlF appearance profiles.

To test whether the observed locations of the rising edges of the AlF appearance profiles are compatible with a Maxwell-Boltzmann distribution of speeds (Eqn 1 of the main text) for AlF leaving the IL surface, the profiles were forward-predicted on the basis of Eqn 2 of the main text, *i.e.*

$$P_t(t)\,dt \propto \frac{z^3}{(t-t_0)^4}\exp\left(\frac{-mz^2}{2kT(t-t_0)^2}\right)dt \qquad \text{Eqn (S7)}$$

The distance, $z$, was taken to be the series of values in Figure 9 of the main text. Since the earliest signals in the AlF profiles result from reaction of $Al^+$, as is apparent from Figure 8 in the main text, $t_0$ was set to the peak arrival time ( = 7 μs) of the relatively narrow $Al^+$ packet at the IL surface (see Figure 8 b) of the main text). The mass was fixed for AlF and the temperature was that of the IL (320 K).

The results are shown in Figure S8. Clearly, the absolute location of the rising edge, and its dependence on distance is predicted accurately, with no adjustable parameters. This reinforces the conclusion, reached separately based on the propagation of the peak of the profile (Figure 9 of the main text), of predominantly thermal-like AlF speeds.

We have not attempted to simulate the additional broadening that is apparent in Figure S8. Note that some of it, particularly later in the profile, is the result of the additional component of AlF from reaction of Al, which has a broader distribution of arrival times (see Figure 8 c) of the main text). The narrower width of the $Al^+$ incident distribution (Figure 8 b) of the main text) is not sufficient, though, to explain the observed width of the AlF profile between its rising edge and peak. We believe that this is primarily the result of the spread in pathlengths between different points within the dosed area of the IL surface and along the finite length of the LIF probe volume,

neither of whose dimensions are negligible relative to the perpendicular distance between the surface and the probe position.

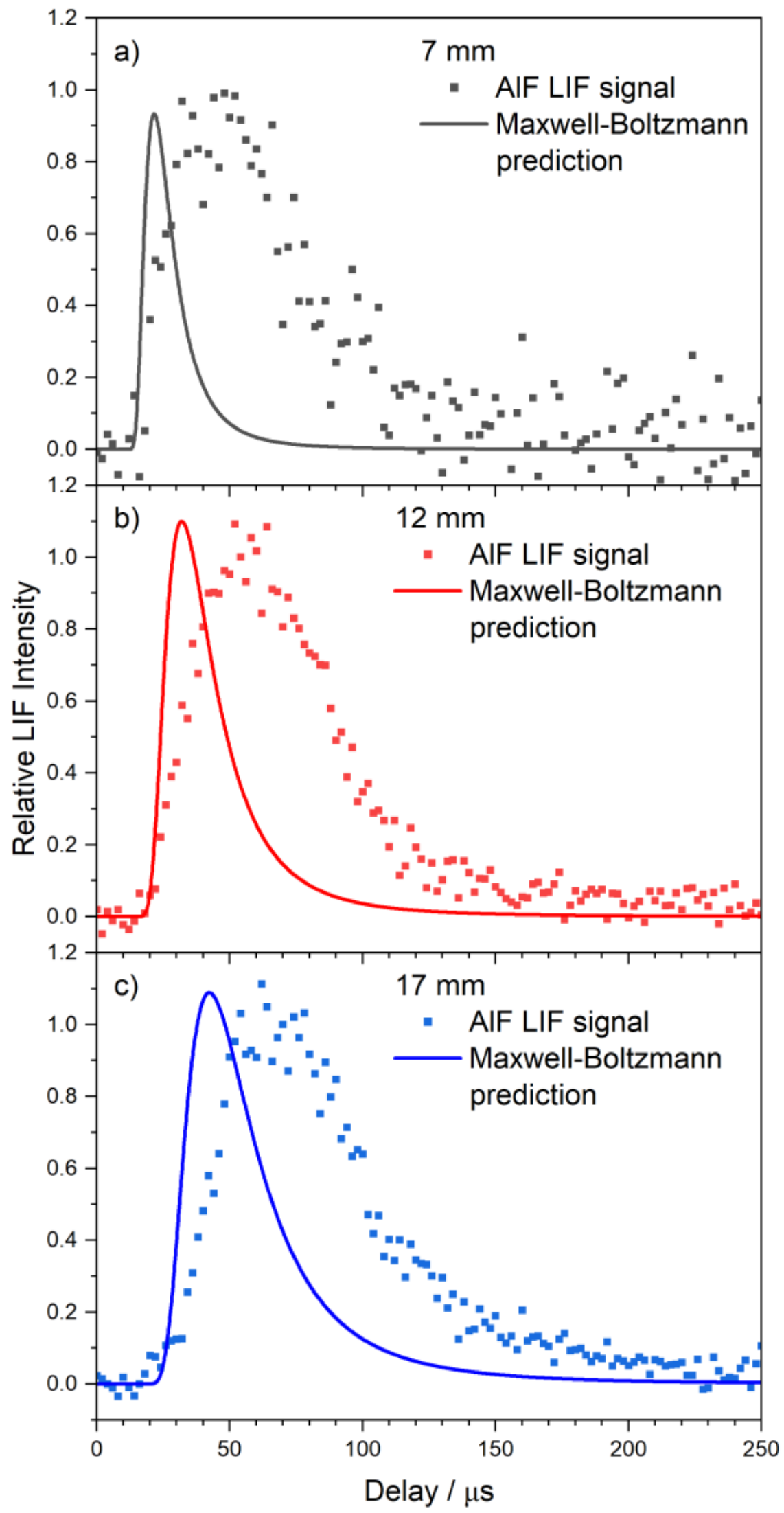


Figure S8: AlF LIF appearance profiles at different IL surface-to-probe distances, as indicated, with corresponding Maxwell-Boltzmann predictions (see Eqn S7) overlaid. There are no adjustable parameters; the fixed values were $m$ = 45.98 amu; $T$ = 320 K; and $t_0$ = 7 µs, corresponding to the most probable TOF of the $Al^+$ at the IL surface. Each trace is separately normalized to the peak of the observed LIF signal.

## S8. References for Supporting Information

(1) Duncan, M. A. Spectroscopy of metal ion complexes: Gas-phase models for solvation. *Annu. Rev. Phys. Chem.* **1997**, *48*, 69–93.
(2) Iskra, A.; Gentleman, A. S.; Kartouzian, A.; Kent, M. J.; Sharp, A. P.; Mackenzie, S. R. Infrared Spectroscopy of Gas-Phase $M^+(CO_2)_n$ (M = Co, Rh, Ir) Ion-Molecule Complexes. *J. Phys. Chem. A* **2017**, *121*, 133–140.
(3) Cunningham, E. M.; Gentleman, A. S.; Beardsmore, P. W.; Mackenzie, S. R. Infrared spectroscopy of closed s-shell gas-phase $M^+(N_2O)_n$ (M = Li, Al) ion-molecule complexes. *Mol. Phys.* **2019**, *117*, 2990–3000.
(4) Brewer, E. I.; Green, A. E.; Gentleman, A. S.; Beardsmore, P. W.; Pearcy, P. A. J.; Meizyte, G.; Pickering, J.; Mackenzie, S. R. An infrared study of $CO_2$ activation by holmium ions, $Ho^+$ and $HoO^+$. *Phys. Chem. Chem. Phys.* **2022**, *24*, 22716–22723.
(5) Wiley, W. C.; McLaren, I. H. Time-of-Flight Mass Spectrometer with Improved Resolution. *Rev. Sci. Instrum.* **1955**, *26*, 1150–1157, Article.
(6) Lane, P. D.; Elstone, N. S.; Bruce, D. W.; Slattery, J. M.; Costen, M. L.; McKendrick, K. G. Detection of Different Classes of Fluorinated Anions at Ionic-Liquid Surfaces by Reactive-Atom Scattering Using Laser-Ablated Al Projectiles. *J. Phys. Chem. C* **2026**, *130*, 2975–2987.
